# Aging microenvironment and antitumor immunity for geriatric oncology: the landscape and future implications

**DOI:** 10.1186/s13045-023-01426-4

**Published:** 2023-03-21

**Authors:** Binghao Zhao, Bo Wu, Nan Feng, Xiang Zhang, Xin Zhang, Yiping Wei, Wenxiong Zhang

**Affiliations:** 1 Department of Thoracic Surgery, The Second Affiliated Hospital of Nanchang University, Nanchang University, 1 Minde Road, Nanchang, 330006 China; 2 Departments of Neurosurgery, Peking Union Medical College Hospital, Chinese Academy of Medical Sciences and Peking Union Medical College, Beijing, 100032 China; 3 Jiangxi Medical College, Nanchang University, Nanchang, 330006 China

**Keywords:** Senescent, Geriatric cancer, Tumor microenvironment, Immunotherapy

## Abstract

The tumor microenvironment (TME) has been extensively investigated; however, it is complex and remains unclear, especially in elderly patients. Senescence is a cellular response to a variety of stress signals, which is characterized by stable arrest of the cell cycle and major changes in cell morphology and physiology. To the best of our knowledge, senescence leads to consistent arrest of tumor cells and remodeling of the tumor-immune microenvironment (TIME) by activating a set of pleiotropic cytokines, chemokines, growth factors, and proteinases, which constitute the senescence-associated secretory phenotype (SASP). On the one hand, the SASP promotes antitumor immunity, which enhances treatment efficacy; on the other hand, the SASP increases immunosuppressive cell infiltration, including myeloid-derived suppressor cells (MDSCs), regulatory T cells (Tregs), M2 macrophages, and N2 neutrophils, contributing to TIME suppression. Therefore, a deeper understanding of the regulation of the SASP and components contributing to robust antitumor immunity in elderly individuals with different cancer types and the available therapies is necessary to control tumor cell senescence and provide greater clinical benefits to patients. In this review, we summarize the key biological functions mediated by cytokines and intercellular interactions and significant components of the TME landscape, which influence the immunotherapy response in geriatric oncology. Furthermore, we summarize recent advances in clinical practices targeting TME components and discuss potential senescent TME targets.

## Introduction

In the traditional view, tumors cause a disease that is closely associated with age. However, from the perspective of cellular function, senescent cells and tumor cells have diametrically opposite behavior. Senescent cells manifest a loss of function or cessation of proliferation, while tumors manifest hyperproliferation and increased metabolism rates [1]. Recently, many studies have shown that aging involves multiple mechanisms, which both prevent cancer and promote tumorigenesis [2]. Therefore, the association between aging and tumors needs to be further explored. In addition, in elderly individuals, the cellular microenvironment is changing, which in turn allows tumors to survive in a unique cytokine, extracellular matrix (ECM), and vascular environment. This specific microenvironment contributes to tumor growth and cancer cell invasion and immune escape [3]. Senescence is a damage-induced cellular response to cancer treatment. Leonard Hayflick and colleagues first observed that human diploid fibroblasts undergo a finite number of doublings before irreversibly arresting, the process named senescence [4–6]. Senescence indicates a conserved response to many different types of external and internal cellular stress, including telomere shortening and oncogenic, genotoxic, metabolic, and oxidative damage, and the instances of all these responses can increase during cancer therapy [7–9]. Senescence has been revealed to be a broad physiological response to tissue damage that plays a pleiotropic role in aging, embryonic development, wound healing, tissue regeneration, and, importantly, responses to oncogenesis and cancer therapy [10–15].

**Fig. 1:**
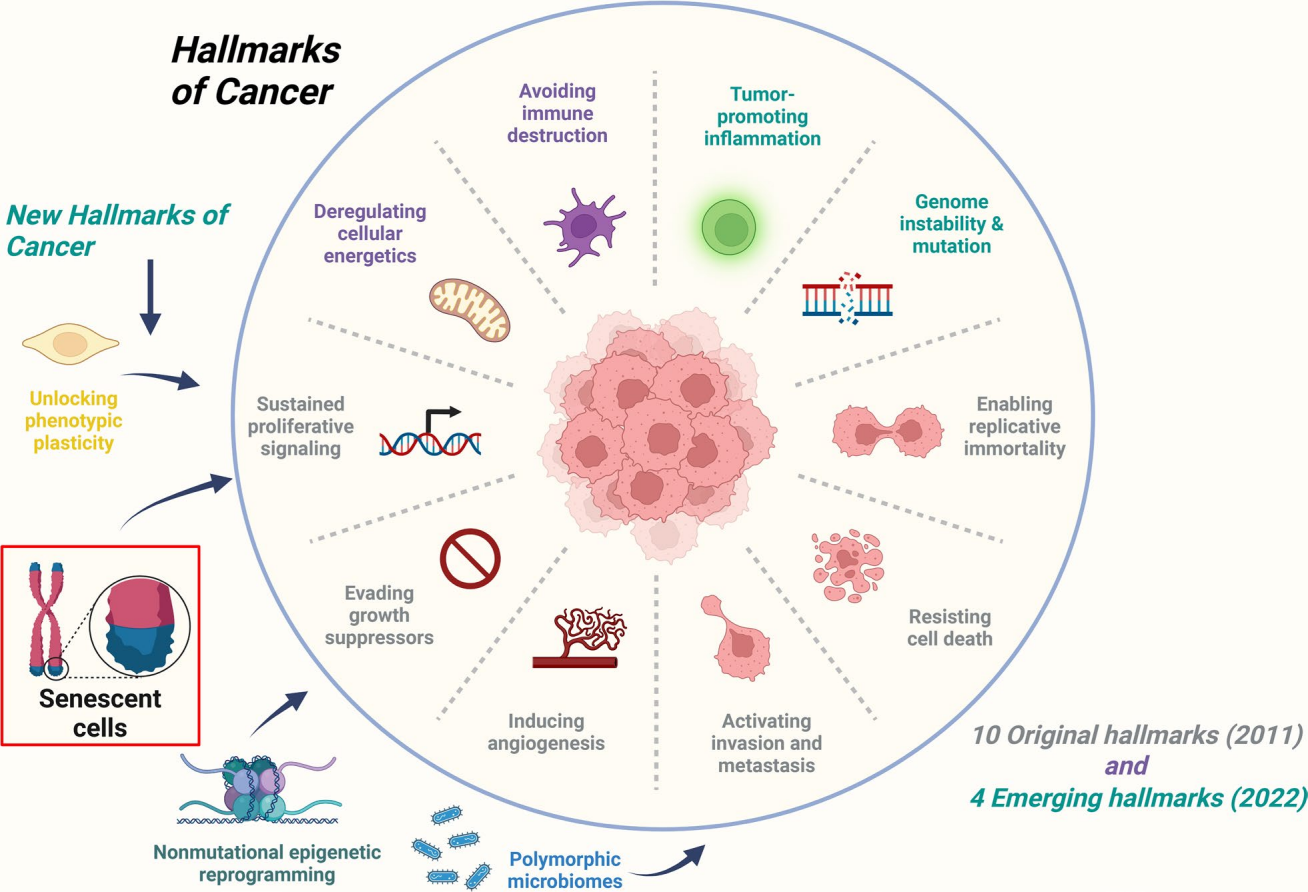
The hallmarks of cancer and new additions. Besides the original hallmarks of cancer, four new hallmarks of “senescent cells,” “unlocking phenotypic plasticity,” “nonmutational epigenetic reprogramming,” and “polymorphic microbiomes” have been added to these parameters. The four new hallmarks are believed to stimulate debate, discussion and experimental elaboration, also give us multiple facets of understanding on human cancer. Cancer senescence can be regarded as a distinct TME feature as it leads to further research interest. TME, tumor microenvironment

The mechanisms of both cancer and aging are based on a time-dependent accumulation of cell damage. Previous studies have shown that many of the hallmarks of aging, including epigenetic alterations, intracellular interaction changes, changes in protein homeostasis (proteostasis), mitochondrial dysfunction and molecular senescence, are common to cancer [16]. In 2020, cancer contributed to 18% of all deaths and remained the second leading cause of death after heart diseases in the USA. However, it is the leading cause of death among women aged 40–79 years and men aged 60–79 years. In the USA, from 2017 to 2019, the probability of developing invasive cancer is 34% in 70 years and older male populations and 27.2% in female populations. In 2019, there were approximately 140,690 new cancer cases diagnosed and 103,250 cancer deaths among the “oldest old” (≥ 85 yr), also the cancer incidence rates peaked in the oldest men and women in 1990 although the rates have subsequently declined. Based on these statistics, a considerable number of cancer cells acquire aging phenotypes [17–19]. Many studies have highlighted that aging can markedly affect normal cells in the tumor microenvironment (TME) and thus promote tumor progression and metastasis. Fibroblasts and immune cells are thought to play necessary roles in this age-related impact [20, 21]. Tumor progression often requires genetic mutations in growth pathway genes to drive hyperproliferation, and distinct mutations trigger senescence biological processes. Aging is associated with many factors that are involved in the aforementioned processes, such as enhanced genomic damage (point mutations, deletions, and translocations), telomere attrition, epigenetic alternation, impaired proteostasis, and deregulation of nutrient sensing [22, 23]. Many environmental factors, such as ultraviolet radiation exposure, alcohol, smoking, and pollution, contribute to the chronic accumulation of DNA damage and other events associated with cellular aging. Previous studies suggested that the cellular aging process of somatic selection is nonautonomous and is, in fact, defined by TME-imposed increases in positive selection for previously accumulated genetic and/or phenotypic diverse senescent tissues, which is leveraged to ensure that senescent models can induce cancer across tissues and species [24]. Many factors involved in senescent tissue evolution result in the final transformation to malignancy and hyperplastic growth in self-renewing tissues, which contribute to growth arrest, apoptosis, and the degradation of other cells and structural tissue components. With increasing age, the cancer risk and many degradative features within tissues and cells exponentially increase [23]. An increasing number of studies have focused on the complex interrelationship between an aged local and systematic TME and its fundamental role in tumor development and progression (Fig. 1). In addition, age-induced reprogramming of stromal components in an established TME seems to contribute to tumor metastasis and progression. Interestingly, clear impact of senescent stromal cells on cancer outcomes is not determined yet or even contradictory, which suggests that different stromal tissue environments in the body may be reprogrammed differently during the aging process. This mechanism ultimately influences tumor growth and progression with respect to the original tissue [25–27]. In this review, we will (1) discuss the interactions between tumors and an aged TME, identifying how TME changes during aging facilitate the reprogramming of stromal cell populations, the ECM, and immune cell infiltration to initiate cancer and progression; (2) then investigate how an aging TME regulates the potential responses of cancer cells to chemotherapy, radiotherapy, targeted therapy, and immunotherapies; and (3) summarize recent clinical progress in geriatric oncology and aging TME. With these foci, we hope to identify additional tumor therapy methods for this special population of aging individuals.

### Intracellular changes in senescent cells

Increased levels of transcription and protein synthesis in senescent cells converge to promote a distinct aspect of senescence, the acquisition of the senescence-associated secretory phenotype (SASP), which promotes senescent cells to communicate damage signals with neighboring cells, including immune cells, fibroblasts, endothelial cells, and adjacent nontumor epithelial cells in the TME in a nonautonomous manner in cells [28, 29] (Table 1). Enhancer regions of hundreds of SASP factors are made accessible, transcribed by NF-κB and other factors, and translated in an mTOR-dependent manner, contributing to the robust secretion of inflammatory cytokines and chemokines and angiogenic, growth, and ECM-degrading signals [21]. The SASP is also associated with the upregulation of cell surface molecules that modulate the interactions between senescent cells and the immune system [30, 31]. Notably, hallmarks of senescence are neither specific nor universal to all types of senescent cells and that multiple markers are necessary to distinguish senescence from other biological outcomes of cancer therapy [7].

**Table 1:** Age-related immune phenotypes and potential therapeutic consequences

Aging type	Changes in phenotypes	Potential consequences
Innate immunity	(1) decrease chemotaxis; (2) decrease phagocytosis of debris; (3) decrease antigen presentation ability	(1) inhibit the activation of tumor-specific CD8 + cytotoxic T cells; (2) decrease the diversity of tumor-specific CD8 + cytotoxic T cells
Immunosenescence		
B cells	(1) increase precursor cells; (2) increase proinflammatory B cells (TNF-α +): (3) inhibit antibody production, diversity and avidity	(1) decrease antibody production and diversity; (2) inhibit response to novel tumor antigens; (3) promote inflammation and autoantibody production; (4) increase the possibility of immune-related AEs
T cells	(1) increase naive T cells; (2) increase Tregs; (3) decrease T-cell repertoire	(1) decrease recognition and responses to novel tumor antigen; (2) increase antigen recall due to large amount of memory T cells
Bone marrow	(1) increase myeloid lymphoid progenitors; (2) inhibit B-cell maturation	decrease potential adoptive immune response to novel tumor antigens
Thymus	(1) increase epithelial cell attrition; (2) reduce IL-7; (3) decrease mature T-cell production	(1) decrease the amount of naive T cells; (2) decrease the possibility to recognize novel tumor antigens
Cellular senescence	(1) decrease telomere length; (2) inhibit cell proliferation; (3) increase expression of cell cycle inhibitors (p16INK4a and P21CIP/KIP); (4) increase proinflammatory cytokine and matrix remodeling factors	(1) decrease the amount of responding B and T cells; (2) promote cancer metastasis; (3) increase the recruitment of immunosuppressive cells (Tregs, MDSCs)
Inflammaging	(1) increase chronic inflammation; (2) increase the level of IL-6, IL-8, IL-18, TNF-α, CRP	(1) increase tumor mutagenesis via inflammatory mediators; (2) inhibit cytokine production in response to tumor antigens

*AEs* adverse events, *Tregs* regulatory T cells, *MDSCs* myeloid-derived suppressor cells, *IL* interleukin, *TNF* tumor necrosis factor, *CRP* C-reactive protein

**Fig. 2:**
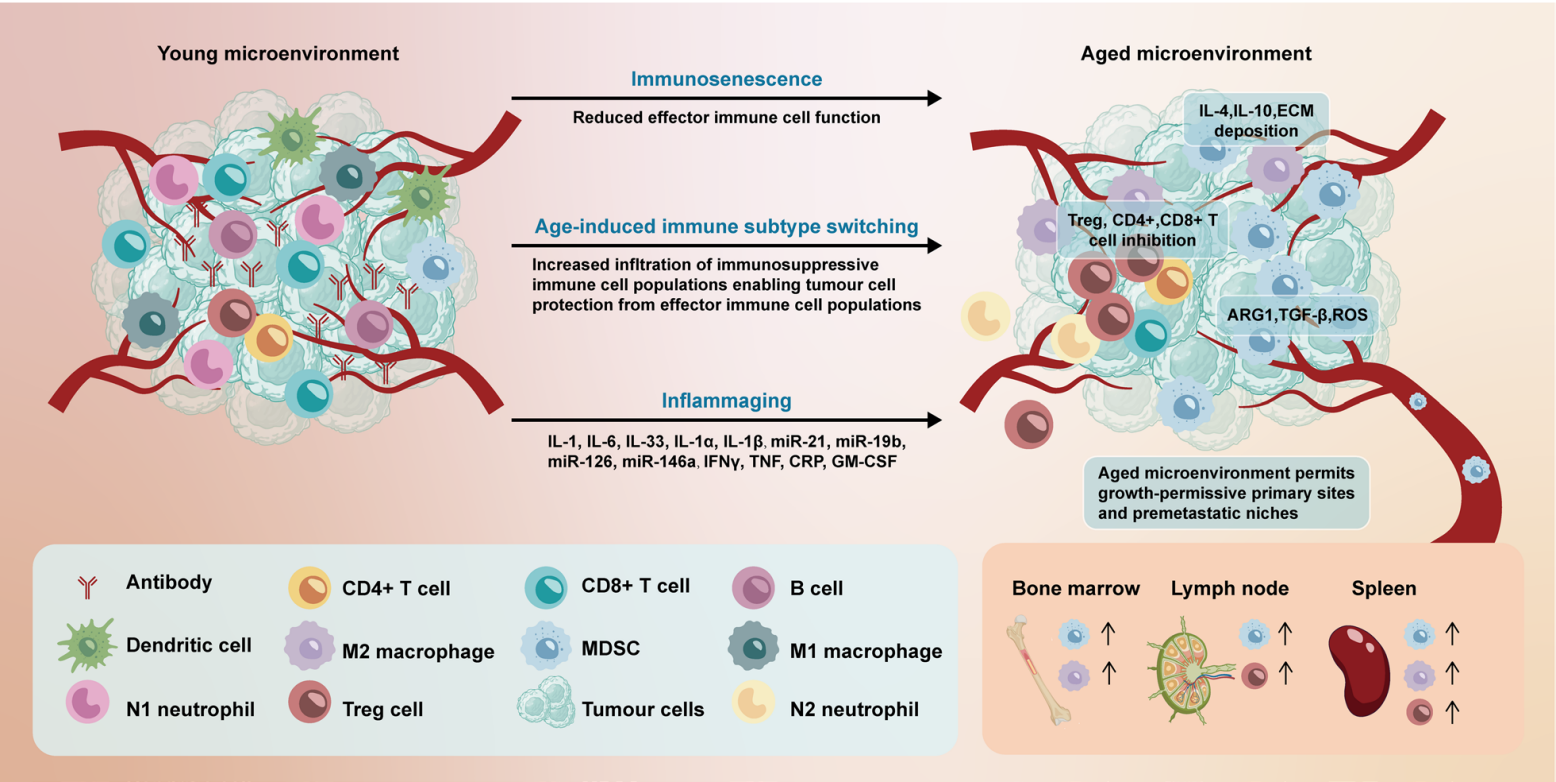
Differences between young and aged TME and senescence-induced factors. One of the critical factors involved in age-related pathologies is immunosenescence defined as a significant decline in overall immune function. The subpopulations of effector immune cells including T cells, NK cells, macrophages, and DCs exhibit a dramatic decrease in cytotoxic activity during the senescence. Age-related immunosenescence plays a key role in promoting tumor formation and accumulation of SASP-secreting cells; the SASP-related decreases in effector immune cell function can also induce tissue-specific switching toward more immunosuppressive cell populations. In the elderly, immunosuppressive MDSCs and Tregs are significantly increased in aged tissue and blood; besides, neutrophils and macrophages appear to switch phenotypically toward immunosuppressive N2 and M2 states, both of which have been shown to promote tumorigenesis of various cancer types, while more direct evidence on the involvement in age-related tumorigenesis is warranted. The accumulation of SASP stromal components results in inflammaging that disrupts acute inflammatory response toward malignant tissue, induces infiltration of immunosuppressive MDSCs and Tregs, and improves secretion of anti-inflammatory components such as cytokines, chemokines, and inflamma-microRNAs. Inflammaging seems to downregulate antitumor activity in aged tissues. NK cells, natural killer cells; DCs, dendritic cells; SASP, senescence-associated secretory phenotype; MDSCs, myeloid-derived suppressor cells; Tregs, regulatory T cells; ARG1, arginase 1; CRP, C-reactive protein; ECM, extracellular matrix; GM-CSF, granulocyte–macrophage colony-stimulating factor; IFN-γ, interferon-γ; IL, interleukin; ROS, reactive oxygen species; TGF-β, transforming growth factor-β; TNF, tumor necrosis factor (Mainly from 10.1038/s41568-019-0222-9 [3])

The dynamic nature of the SASP and its ability to modulate the surrounding tissue TME and immune responses in different ways is thought to be associated with many contrasting physiological characteristics of senescence (Fig. 2 and Table 2). In fact, senescence can lead to anti- or protumorigenic outcomes depending on the senescence inducer, the duration of the senescent period, the SASP factors produced, and the tissue and disease context [32, 33]. Biomarkers of cellular senescence have been thoroughly investigated in precancerous tissues in different solid organs in humans, including the lungs, prostate, pancreas, and skin, and have been found to be lost during neoplastic progression [15, 34]. Therefore, it has been postulated that senescence may suppress tumors and thus block tumor development by preventing the proliferation of potentially malignant cells. Supporting this hypothesis, oncogene-induced senescence following aberrant RAS activation led to the arrest of premalignant cells and secretion of proinflammatory SASP factors that promoted innate and adaptive immunity cell clearance of incipient cancer cells and blocked tumor formation [35]. Similarly, therapy-induced senescence (TIS) has been shown to inhibit tumor growth and lead to an influx of cytotoxic CD8+ T cells and natural killer (NK) cells that promote tumor regression [36, 37]. In contrast, some evidence has demonstrated that the SASP after chemotherapy can promote tumors through the secretion of immune suppressive factors and attraction of immune suppressive cells, as well as the production of angiogenic and other growth factors, which enhance the invasion and metastasis of adjacent non-senescent tumor cells [38, 39]. Senescence is generally presumed to be neither a permanent nor irreversible state and tumor cells that bypass senescence through the acquisition of genomic instability (e.g., polyploidy) likely achieve enhanced stemness and tumorigenic potential that contributes to drug resistance and tumor relapse [40–43].

**Table 2:** The role of SASP in tumor microenvironment

	Cancer type	Senescent cell	Senescence inducer	Major roles of SASP	SASP factors
Protumorigenic SASP	hepatocyte	hepatocyte	OIS (N-Ras)	(1) myeloid cell recruitment; (2) MDSC differentiation	CCL2
	hepatocyte	hepatic stellate cell	HFD	antitumor immunity of CD8+ T cells impairment	PGE2
	lymphocyte	lymphocyte	TIS (doxorubicin)	stemness induction	not reported
	mammary epithelial cell	mammary epithelial cell	TIS (doxorubicin)	mitogenic support	Eotaxin, CXCL5, Rantes
	mammary epithelial cell	fibroblast	DNA damage (bleomycin)	cancer invasion promotion	MMPs
	mammary epithelial cell	mammary epithelial cell	OIS (HER2)	cancer metastasis promotion	not reported
	melanocyte	fibroblast	TIS (CDK4/6 inhibitor)	myeloid cell recruitment	not reported
	mesothelial cell	mesothelial cell	TIS (pemetrexed)	(1) EMT induction; (2) chemoresistance	not reported
	prostate epithelial cell	prostate epithelial cell	TIS (PTEN loss)	myeloid cell recruitment	CXCL1, CXCL2
	prostate epithelial cell	prostate epithelial cell	TIS (PTEN loss)	MDSC recruitment	not reported
	thyroid follicular cell	thyroid follicular cell	OIS (BRAF)	anoikis resistance	CXCL12
Antitumorigenic SASP	hepatocyte	hepatocyte	OIS (N-Ras)	immune-related senescent cell clearance	IL-1α
	lymphocyte	lymphocyte	TIS (cyclophosphamide)	cellular senescence reinforcement	not reported
	melanocyte	melanocyte	TIS (AURKA or CDK4/6 inhibitor)	lymphocyte recruitment	CCL5
	melanocyte	melanocyte	TIS (Aurora inhibitor)	cellular senescence reinforcement	not reported
	osteoblast	osteoblast	TIS (radiotherapy)	NKT cell recruitment	IL-6
	pancreatic ductal cell	pancreatic ductal cell	TIS (MEK and CDK4/6 inhibitors)	(1) vascularization promotion (2) drug delivery improvement (3) endothelial cell activation (4) CD8+ T-cell accumulation	VEGF, CCL5, CXCL1, IL-6
	hepatocyte	hepatocyte	OIS (N-Ras)	(1) myeloid cell recruitment (2) macrophage differentiation	CCL2

*SASP* senescence-associated secretory phenotype, *OIS* oncogene-induced senescence, *HFD* high-fat diet, *TIS* therapy-induced senescence, *EMT* epithelial–mesenchymal transition, *MDSC* myeloid-derived suppressor cells, *NKT* natural killer T cell (Mainly from 10.1002/1878-0261.13268)

During aging, cells undergo a series of intracellular changes. In the nucleus, during cell division and senescence, telomeres at the ends of DNA sequences are shortened, resulting in their reduced binding to protective protein complexes, which protect DNA from DNA damage response (DDR) factors. Shortened telomeres and DDR activation may establish connections between senescent cells and tumor cells [44]. Telomeres act a necessary role in protecting chromosome ends, preventing DDR, and maintaining genomic stability. There are two telomere maintenance mechanisms (TMMs) in human cancer to keep the infinite capacity for tumor proliferation: one is telomerase-mediated maintenance (observed in 85%) and the other is alternative lengthening of telomeres (ALT) (observed in 15%). Unique characteristics of ALT include very long telomeres, telomere length heterogeneity, abundant extrachromosomal linear and circular telomere DNA, increased telomere-sister chromatid exchange (T-SCE) events, and the formation of ALT-associated promyelocytic leukemia (PML) bodies. It is crucial to understand the molecular mechanism underlying ALT and its impact on cancer prognosis as ALT can be therapeutic target [44, 45]. Similarly, with aging, genetic mutations gradually accumulate, especially when cells are exposed to tobacco and other chemicals, ultraviolet rays, ionizing radiation, or exogenous mutagens. Mutation ultimately increases the probability of cancer occurrence. This theory has been verified by cancer driver mutations detected in many middle-aged and older individuals [46]. Moreover, p16, a tumor suppressor protein that regulates Rb protein phosphorylation, accumulates with aging in most mammals and ultimately participates in tumorigenesis [47]. Furthermore, another classic tumor suppressor, p53, has been shown to induce senescence by affecting the downstream factor p51 [48]. In addition to changes in DNA and gene expression, the epigenetic inheritance of senescent cells regulates tumor formation and progression. The rate of methylation, a common gene repressive modification, increases with age. Therefore, methylation of tumor suppressor gene promoters, such as the VHL promoter, has been suggested to contribute to angiogenesis, thereby promoting tumors, and hypermethylated cells prefer to undergo oncogenic transformation [49, 50].

In addition to changes at the genetic level, cytoplasmic alterations are involved in aging and tumors. Reactive oxygen species (ROS) are byproducts of mitochondrial electron transfer in aerobic cells. High levels of ROS lead to cell damage and increase genomic instability to induce oncogenic functions [51]. Studies have shown that during aging, dysfunctional mitochondria gradually accumulate, and deleterious events, including membrane potential reduction and proton leakage, eventually lead to increased ROS levels [7]. Moreover, MAPK, PI3K, and STAT3 can be regulated by ROS, which promotes cell proliferation and survival, as has been discovered in breast cancer, lung cancer, pancreatic cancer, and other malignant tumors [52]. In addition, changes in mitochondria cause corresponding changes in AMP/ATP, AMP/ATP, and NAD+/NADH ratios, leading to cell cycle arrest, NF-κB activation, and other changes that are considered to be important to tumor formation [7]. In addition, the endoplasmic reticulum is an important subcellular organelle in lipid synthesis and protein synthesis, and the unfolded protein response (UPR) is triggered when excessive ROS levels cause protein misfolding. In normal senescent cells, the UPR drives cell death, but in breast cancer, the UPR prevents cell death and promotes cancer cell immortality [53]. In addition, centrosome dysfunction may regulate aging, tumorigenesis, and tumor immunity [54].

In addition, the role of the cGAS-STING signaling pathway, constituted by cyclic GMP-AMP synthase (cGAS) and stimulator of interferon genes (STING), has been extensively studied. cGAS recognizes DNA in the cytoplasm and activates IFN expression and NF-κB through multiple cascade reactions. The effects of cGAS-STING on tumors are diverse. On the one hand, the interferon produced by short-term cGAS-STING activation can recruit dendritic cells and CD8+ T cells and promote their maturation. After tumor cells are killed, the tumor-associated antigens that are released are by surrounding DCs, which will further enhance the immune response, thus forming a positive feedback mechanism [55]. A positive correlation between cGAS expression and survival has been reported in human lung adenocarcinoma patients [56]. On the other hand, long-term cGAS-STING activation may promote tumorigenesis. cGAS-STING can assist in the formation of the SASP, ultimately promoting the epithelial–mesenchymal transition (EMT), tumor progression, and invasion [57]. Another study demonstrated that activation of STING might disrupt calcium homeostasis in T cells, leading to cell death [58]. De Cecco found that senescent cells, loss of the nuclear lamin protein Lamin B1 and chromatin fragments located in the cytoplasm can activate the cGAS-STING pathway. In the same study, high activity of long-interspersed element-1 (LINE-1) reverse-transcribes mRNA into cDNA and activates cGAS-STING [59]. Additionally, infection with various DNA viruses, such as human cytomegalovirus and hepatitis B virus, which are common infections in elderly individuals, can activate the cGAS/STING pathway [60]. This process, in turn, modulates the tumor microenvironment in elderly individuals. The aforementioned evidence suggests that cells engage in unique mechanisms to inhibit tumor formation during the early stages of aging and that when inactivated or mutated, these mechanisms may in turn promote tumor formation and progression.

The SASP can reinforce senescent growth arrest and/or promote immune surveillance to suppress cancer [21]. Oncogene-induced and therapy-induced senescent cells secrete the inflammatory cytokine IL-1α, which is a crucial SASP initiator and regulator [61]. IL-1α facilitates an autocrine inflammatory response through the activation of NF-κB, which leads to the transcription of IL-6 and IL-8 [61]. Subsequently, these inflammatory cytokines reinforce senescence-related proliferation arrest through the increased production of reactive oxygen species and a sustained DNA damage response, particularly in oncogene-induced senescent cells [61, 62]. In addition, IL-1α mediates paracrine senescence in neighboring cells to suppress tumor progression [63], and IL-1α, IL-6, and IL-8 mediate the recruitment of M1-like macrophages, T helper 1 cells, and NKs to the TME. Infiltrative immune cells drive the elimination of senescent tumor cells and may also eliminate non-senescent cancer cells via a bystander effect [35, 64]. Some immune cells, such as T helper 1 cells, can also trigger senescence in cancer cells through the secretion of inflammatory cytokines [65]. The SASP of senescent cancer cells is thought to initially suppress tumorigenesis but to be mostly detrimental in the long term [38, 66]. In an in vivo study, proliferation and tumorigenesis of both premalignant and malignant epithelial cells were increased when they were coinjected with human senescent fibroblasts into mice [67]. Another study showed that MMPs secreted by senescent human fibroblasts were critical for promoting tumorigenesis [68]. Prominent SASP factors are involved in ECM processing and degradation, which can promote tumor cell proliferation and invasion [69]. Additionally, MMPs promote the release of many other cytokines and growth factors supporting tumorigenesis, such as vascular endothelial growth factor (VEGF), which promotes tumor-driven angiogenesis [70], and the chemokine CXCL1, which promotes tumor growth [71].

IL-6 and IL-8, known SASP-associated factors, mediate the protumorigenic effects of senescent cells because they establish a chronic inflammatory TME that triggers tumor growth [28, 72]. In addition, IL-6 and IL-8 drive the transcription of genes encoding MMPs and drive the epithelial-to-mesenchymal transition, thereby promoting tumor invasiveness [73–76]. IL-6 also recruits myeloid-derived suppressor cells (MDSCs) to the TME to mediate the protumorigenic effects of senescent cells, which block IL-1α signaling and antagonize senescence in cancer cells [77, 78]. In addition, MDSCs block immune surveillance by inhibiting CD8+ T cells and NK cells through the actions of IL-6 and CCL2, respectively [64, 78, 79]. However, the protumorigenic and antitumorigenic effects of senescent cancer cells are likely mediated by a comprehensive interaction between multiple SASP factors and the immune TME. Furthermore, the effects of SASP factors are likely impacted by tissue type, residual immune cells, inflammatory networks, and senescence inducers. Therefore, it is difficult to precisely identify whether the effects of senescent cancer cells are protumorigenic or antitumorigenic.

Several studies have demonstrated that senescence-induced therapies are associated with complex reprogramming that ultimately drives stemness in both tumor and normal cells [40, 41]. Moreover, senescent cancer cells that are not eliminated by the immune system can spontaneously resist proliferation arrest under certain circumstances and reenter the cell cycle [40, 80]. A study confirmed that oncogene-induced senescent cells entered the cell cycle, particularly by restoring telomerase activity through the derepression of the telomerase reverse transcriptase (TERT) gene [81]. Senescent cells show WNT-dependent enhanced growth and tumor-initiating potential to resume growth [40]. This senescence-associated stemness results in a highly aggressive nature driven by WNT pathway activation independent of WNT ligand binding via the SASP and is enriched in relapsed tumors [40]. Additionally, the expression of β-catenin in pituitary stem cells provokes the acquisition of a senescence signature and the SASP and induces craniopharyngioma tumors in a paracrine fashion. Importantly, mice with a decreased senescent cell burden and an attenuated SASP response exhibited decreased tumorigenic potential, indicating that the SASP may promote tumor induction [82]. Extracellular vesicles and exosomes, components of the SASP, have also garnered considerable interest in the field of senescence, and small vesicles from senescent cells can promote tumors [83–85]. The complex and often unpredictable role of senescence-inducing therapies is derived from the dual role of the SASP. The effect of the SASP is highly dependent on context and cell type and varies during different stages of cancer progression [21, 38, 86]. Specifically, SASP-mediated immunosuppression promotes tumor growth in later stages of tumor progression, while the SASP is a tumor suppressor in the early stages of tumorigenesis [64]. In addition, TP53 may be involved in determining whether the senescence-induced inflammatory response suppresses or promotes tumorigenesis [87]. In the long term, the SASP of senescent tumors is suggested to be primarily detrimental to neoplastic growth, therapy resistance, immunosuppression, metastasis and angiogenesis [67, 78, 87, 88]. However, senescent cancer cells potentially remain dormant for a long time, evading therapy and posing a risk for tumor relapse [15, 89, 90]. It has also been revealed that many genotoxic chemotherapies lead to debilitating side effects caused by senescence induced in normal tissues. Normal senescent cells remain present in the long term and promote local and systematic inflammation caused by the SASP, which results in or exacerbates chemotherapy side effects [88]. Accordingly, it may be helpful to combine senescence-promoting therapy with senolytic therapy in the context of cancer. In addition to direct targeting of cancer cells by delivering a one-two punch and decreasing the side effects of chemoradiotherapies on normal tissues, senolytics may eliminate incipient preneoplastic senescent cells or other senescent cells in the TME to suppress the detrimental effects of the SASP [66, 72].

### Changes in cytokines and their receptors

**Fig. 3:**
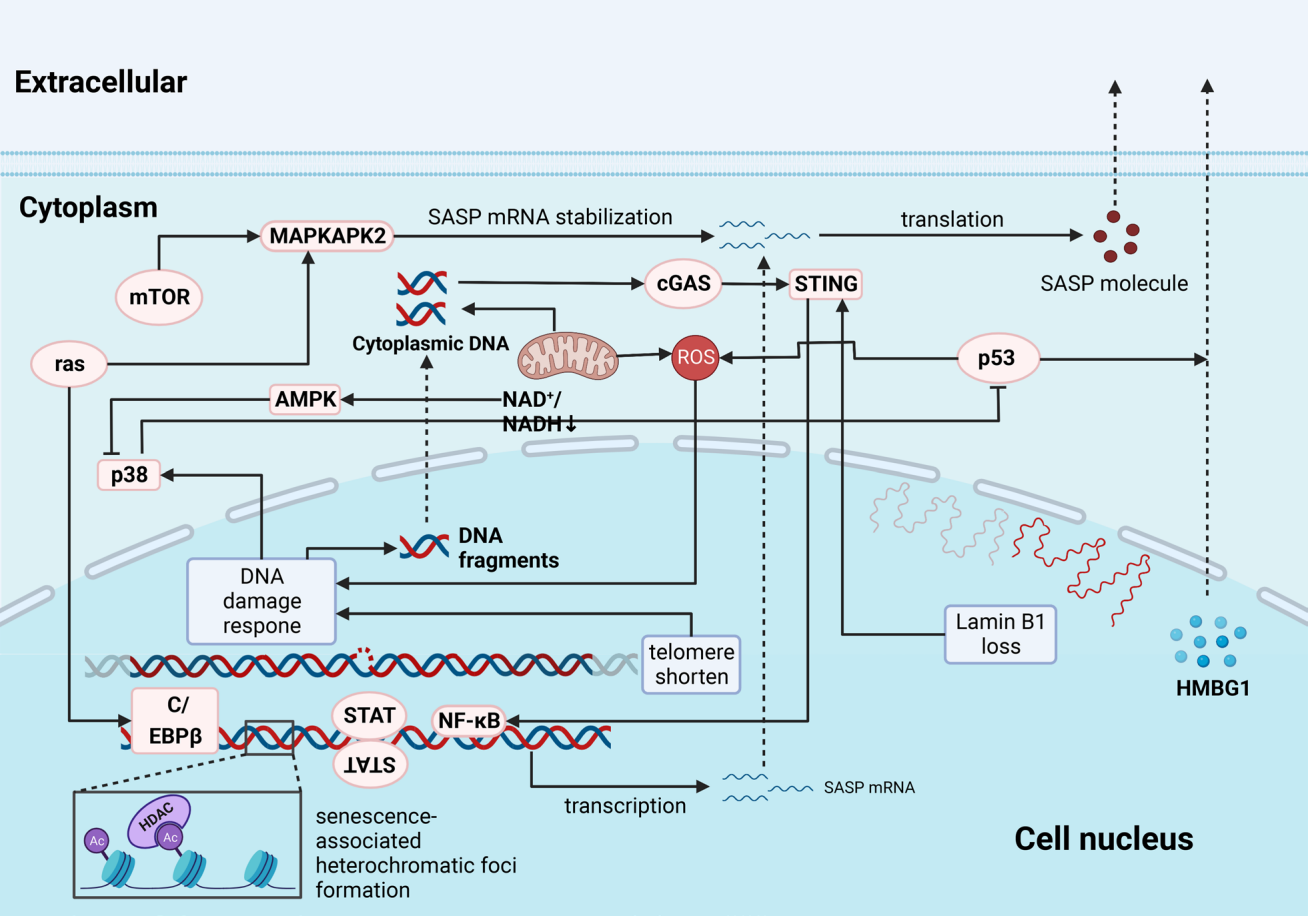
The formation of SASP in senescent cell. The formation of SASP undergoes multiple mechanism and regulator; many time-dependent damage will accelerate the formation of SASP. Actually, transformation of SASP involves many signaling pathways. The deletion of p53 and the upregulated expression of RAS aggravate the paracrine activity of SASP. Besides, the three-dimensional structure of the genome in senescent cells can enhance the SAE activity through the transcription factor C/EBPα/β, thereby promoting the secretion of SASP. DDR-dependent SASP activation accompanies by chromatin remodeling frequently, in which HDAC might be involved. SAE SASP of senescent cells also damages the DNA of adjacent cells and induces senescence, thereby forming senescence-induced senescence. The cytoplasm alternation is also involved in aging and tumors. ROS is a by-product of mitochondrial electron transfer in aerobic cells. High levels of ROS will lead to cell damage and increase genomic instability to exert oncogenic functions. In the process of aging, dysfunction mitochondria will gradually accumulate, and events including membrane potential reduction and proton leakage will occur, eventually leading to increased ROS levels. Not only that, changes in mitochondria will cause corresponding changes in AMP/ATP, AMP/ATP, and NAD + /NADH ratios, thereby leading to cell cycle arrest, NF-κB activation, and other changes that are considered important to tumor formation. Transcribed by NF-κB and other factors, and translated in an mTOR-dependent manner contributing to the robust secretion of SASP-related inflammatory cytokines, chemokines, angiogenic growth, and ECM-degrading signals. SASP, senescence-associated secretory phenotype; DDR, DNA damage response; SAE, senescence activation enhancer; C/EBPα/β, CCAAT/enhancer-binding protein α/β; HDAC, histone deacetylase; ATP, adenosine triphosphate; AMP, adenosine monophosphate

The tumor microenvironment consists of a variety of cytokines that can affect tumor progression, metastasis, and the formation of an immunosuppressive microenvironment. In aging patients, changes in the crosstalk between cytokines and immune cells characterize a unique aging tumor microenvironment [91] (Fig. 3).

#### Formation of senescence and SASP

Cellular senescence serves as a powerful protective mechanism against tumorigenesis [15]. The activation of oncogenes such as *HRAS*^*V12*^ triggers growth arrest, referred to as oncogene-induced senescence (OIS), which was first reported in 1997 [10, 13, 92, 93]. In 2005, the concept of OIS was extended to multiple carcinogenesis models, including lymphomas, prostate cancer, lung adenomas, hyperplastic pituitary gland, and melanocytic nevi [93–97]. Melanocytic nevi induced by BRAF mutations gradually remain senescent for decades, preventing their progression into melanoma [95]. Similarly, the lack of tumor suppressor genes, such as PTEN, can also induce senescence in the primary prostate epithelium, referred to as PTEN loss-induced cellular senescence (PICS) [94]. In older adults, the composition of cytokines and immune cells changes because of SASP acquisition. The acquisition of the SASP involves multiple mechanisms and regulators. Time-dependent damage accelerates SASP acquisition. Several studies have shown that + transformation of the SASP involves many signaling pathways [3]. The deletion of P53 and the upregulated expression of RAS aggravate the paracrine activity of the SASP [28]. Moreover, the three-dimensional structure of the genome in senescent cells enhances senescence activation enhancer (SAE) activity through the action of the transcription factor CCAAT/enhancer-binding protein α (C/EBPα), thereby promoting the secretion of SASP factors [98]. DDR-dependent SASP acquisition is frequently accompanied by chromatin remodeling, in which histone deacetylase (HDAC) might be involved [99]. The SASP in senescent cells also damages the DNA of adjacent cells and induces their senescence, causing senescence-induced senescence [100, 101]. In addition, OIS is mediated by activation of the INK4A-RB pathway, independent of p53 activation and DNA damage signaling [10, 92, 102]. Moreover, senescence can be triggered by other oncogenic pathways, such as the activated MYC pathway, which increases the levels of the ARF-encoding transcript at the CDKN2A locus, resulting in stabilized p53 [103] and hyperactivated WNT-β-catenin signaling, leading to the DNA damage response via the p53-p21 pathway [104–108].

Malignant cells can be forced to enter a senescent state via therapy-induced senescence, and conventional therapeutics such as chemotherapy or radiotherapy show the ability to induce senescence in cancer cells [9, 36, 37, 109–124] (Table 3). In a chemotherapy-induced senescent state, apoptosis is induced when higher doses of drugs are applied [125–127]. Mechanistically, many chemotherapies cause DNA damage in cancer cells, which triggers senescence through ATM-CHK2 and ATR-CHK1 kinase-mediated activation of the interconnected p53-RB pathways [128, 129]. Topoisomerase I and II inhibitors, such as doxorubicin, have been shown to dysregulate the re-ligation of DNA strands after supercoil unwinding, leading to large-scale DNA damage and increasing expression of p53 and its downstream targets CDKN1A and SERPINE1, thereby inducing senescence [130–132]. Platinum-based therapies, including cisplatin, carboplatin, and oxaliplatin, induce DNA damage through DNA cross-linking, leading to senescence induction [133, 134]. Alkylating agents such as temozolomide, dacarbazine, and busulfan cross-link with DNA by reacting with atoms in DNA, triggering a DNA damage-mediated senescence response [135]. Cell cycle dysfunction caused by microtubule inhibitors (paclitaxel and docetaxel) may cause extensive DNA damage and trigger a p53-p21 pathway-facilitated senescence response [136, 137]. Methotrexate and gemcitabine induce genotoxic stress by blocking DNA synthesis, thereby inducing cellular senescence [138, 139]. Radiotherapy is widely used for the treatment of multiple cancer types and can induce irreparable DNA damage response that activates ATM or ATR and p53-p21 pathway-mediated apoptosis and cellular senescence [129, 140, 141]. Since radiotherapy is applied locally, the tissue surrounding a tumor shows an increase in senescent cell burden that results in immunosuppressive effects [38, 78, 142].

**Table 3:** Senescence-induced therapies and immunotherapy response in cancer

Senescence-inducing therapy type	Therapy	Cancer and model	Senescence biomarkers	Potential immune response	Potential tumor response	Reference
Radiotherapy	radiotherapy	osteosarcoma mouse model	SA-β-gal, p16, p21, SASP (IL-6, CCL2/3/4/5)	increase NKT activation ↑	↓	[111]
	radiotherapy	NSCLC human cell line xenografts	SA-β-gal, STING, NF-κB, L1, p21, SASP (IFN-β, IL-1α, IL-6)	increase macrophage activation ↑	↓	[112]
	radiotherapy + PARPi (ex vivo in tumor cells)	(1) melanoma; (2) PDAC syngeneic transplant mouse models	SA-β-gal, p16, p21, SASP (CCL5, IFN-β, CXCL9/10/11)	increase DC, CD8+ T, NK activation ↑	↓	[113]
Chemotherapy	cyclophosphamide	B-cell lymphoma syngeneic transplant mouse model	SA-β-gal, NF-κB, p15, SASP (IL-6, IL-8, ICAM-1, CXCL1)	increase NK activation ↑	↓	[114]
	doxorubicin or melphalan	MM syngeneic transplant mouse model	SA-β-gal, p16, p53, NK ligands (RAE-1, MICA, MULT-1, PVR)	increase NK activation ↑	↓	[115]
	cisplatin + irinotecan (ex vivo in tumor cells)	ovarian cancer syngeneic transplant mouse models	SA-β-gal, STING, p16, yH2AX, SASP (IL-6, VEGFA, GM-CSF)	increase DC, CD8+ T activation ↑	↓	[116]
	docetaxel	PCa GEMM	SA-β-gal, p16, p21, SASP (GM-CSF, CSF-1, IL-10, CCL2, CXCL1/2)	(1) increase MDSC activation ↑; (2) decrease NK, CD8+ T activation ↓	↑	[117]
	mitoxantrone, other agents	(1) PCa human xenografts; (2) PCa clinical samples	SA-β-gal, p16, SASP (IL-6, IL-8, MMPs, AREG), PD-L1	decrease CD8+ T activation ↓	↑	[118]
Aurora kinase inhibitors	MLN8054/MLN8237 (AURKAi)	(1) melanoma human xenografts; (2) PDXs; (3) syngeneic	SA-β-gal, NF-κB, SASP (IL-6, IL-8, CCL5, CXCL1/2)	increase macrophage, CD8+ T activation ↑	↓	[119]
	MLN8237 (AURKAi)	melanoma patient samples	SASP (CCL5)	increase CD8+ T activation ↑	unclear	[119]
	AZD1152 (AURKBi)	(1) melanoma; (2) CRC syngeneic transplant mouse models	SA-β-gal, p21	increase CD8+ T activation ↑	no significant change	[120]
Cell Cycle inhibitors	abemaciclib (CDK4/6i)	(1) ER + breast cancer GEMM; (2) PDXs	SA-β-gal, MHC-I	(1) increase CD8+ T activation ↑; (2) decrease Treg response ↓	↓	[121]
	abemaciclib (CDK4/6i)	melanoma syngeneic transplant mouse models	SA-β-gal, SASP (CCL20, CX3CL1)	release T-cell suppression ↓	no significant change	[122]
	palbociclib (CDK4/6i) + trametinib (MEKi)	LUAD GEMM	SA-β-gal, NF-κB, p15, SASP (TNF-α, ICAM-1, IL-15, NKG2D ligands)	increase NK activation ↑	↓	[36]
	palbociclib (CDK4/6i) + trametinib (MEKi)	PDAC GEMM	SA-β-gal, SASP (VEGFs, PDGFs, MMPs, IL-6, CXCL1, CCL5), MHC-I, PD-L1	increase CD8+ T activation ↑	no significant change	[37]
	palbociclib (CDK4/6i) (ex vivo in fibroblasts)	melanoma syngeneic transplant mouse models	SA-β-gal, NF-κB, p16, SASP (IL-6, MMP3, CCL6, CCL8, CCL11)	increase MDSCs activation ↑	↑	[123]
	XL413 (CDC7i)	(1) HCC GEMM; (2) human xenografts	SA-β-gal, p16	increase Mac, CD8+ T, CD4+ T activation ↑	↓	[124]
Pro-senescence + Immunotherapy	cisplatin + irinotecan (chemotherapy) + a-PD-1 ICI	ovarian cancer syngeneic transplant mouse models	SA-β-gal, STING, p16, yH2AX, SASP (IL-6, VEGFA, GM-CSF)	increase CD8+ T, DC infiltration ↑	↓↓	[116]
	Mitoxantrone (chemotherapy) + a-PD-1 ICI	PCa human xenografts	SA-β-gal, p16, SASP (IL-6, IL-8, MMPS, AREG), PD-L1	increase CD8+ T infiltration ↑	↓	[118]
	MLN8237 (AURKAi) + a-CD137 (T cell agonist)	melanoma syngeneic transplant mouse models	SA-β-gal, NF-κB, SASP (IL-6, IL-8, CCL5, CXCL1/2)	increase CD8+ T infiltration ↑	↓↓	[119]
	AZD1152 (AURKBi) + a-CTLA-4 ICI	(1) melanoma; (2) CRC syngeneic transplant models	SA-β-gal, p21	increase CD8+ T infiltration ↑	↓	[120]
	abemaciclib (CDK4/6i) + a-PD-1 ICI	ER + breast cancer GEMM	SA-β-gal, MHC-I	increase CD8+ T infiltration ↑; decrease Treg activation ↓	↓↓	[121]
	abemaciclib (CDK4/6i) + a-PD-1/CTLA-4 ICI	melanoma syngeneic transplant mouse models	SA-β-gal, SASP (CCL20, CX3CL1)	release T-cell suppression ↓	↓	[122]
	palbociclib (CDK4/6i) + trametinib (MEKi) + a-PD-1 ICI	PDAC GEMM	SA-β-gal, NF-κB, SASP (VEGFs, MMPs, PDGFs, IL-6, CXCL1, CCL5), MHC-I, PD-L1	increase CD8+ T activation ↑	↓	[123]

*SA-β-gal* senescence-associated beta-galactosidase, *SASP* senescence-associated secretory phenotype, *NSCLC* non-small cell lung cancer, *PDAC* pancreatic ductal adenocarcinoma, *NKT* Natural Killer T cell, *DC* dendritic cell, *NK* Natural Killer cell, *PCa* prostate cancer, *GEMM* genetically engineered mouse model, *MDSC* myeloid-derived suppressor cell, *AURKA* Aurora Kinase A, *AURKB* Aurora Kinase B, *PDX* patient-derived xenograft, *CRC* colorectal cancer, *ER* estrogen receptor, *HCC* hepatocellular carcinoma, *ICI* immune checkpoint inhibitor, *LUAD* lung adenocarcinoma, *MM* multiple myeloma, *i* inhibitor (Mainly from 10.1016/j.semcancer.2022.02.005)

Upregulation of cyclin-dependent kinase (CDK) inhibitor proteins such as INK4A and p21 to induce cell cycle arrest is a hallmark of senescent cells [143]. CDK4/6 are important for the progression from the G1 phase to the S phase of the cell cycle and are overexpressed in a number of human cancers. CDK4/6 mimic the function of INK4A and induce senescence in various cancer cells [144–151]. A triple CDK2/4/6 inhibitor (PF-06873600) that is still being investigated for the treatment of breast cancer has been shown to be a potential senescence inducer in various cancer models [152, 153]. Inhibition of DNA replication through small-molecule inhibition of the kinase CDC7, for example, by XL413 or TAK-931, leads to senescence induction in liver cancer cells. This senescence response has been observed only in TP53-mutant tumors, presumably because TP53-mutant tumors retain the ability to be arrested in the cell cycle upon CDC7 inhibition [124].

Numerous compounds inhibiting telomerase complex action have been identified as candidates for anticancer therapy [154], among which BIBR15 and GRN163L are potent telomerase inhibitors that greatly promote senescence and suppress cancer cell proliferation [155–157]. However, the use of GRN16 for senescence-promoting therapy should be examined further, as it also induces apoptosis in pancreatic cancer cells [157]. Vorinostat, a histone deacetylase inhibitor, upregulates the expression of multiple tumor suppressor genes, such as CDKN2A and TP53, and induces senescence via these two major pathways in various cancer cell lines [158, 159]. In mouse models, genetic restoration of Trp53 resulted in the regression of sarcomas and liver carcinomas by inducing a senescence response, and an apoptotic response was observed in lymphoma regression [160–162]. Additionally, senescence induction has been shown to be accompanied by the acquisition of the SASP and recruitment of immune cells into tumors, suggesting efficient clearance of senescent cancer cells [160]. The MDM2 inhibitors nutlin-3 and RG7112 interact with p53-MDM2 and show promising results for inducing senescence in tumors retaining wild-type TP53 in human cancer cell models [163–165]. Inactivation of PTEN shows the potential for use in senescence-promoting cancer therapy in vitro and in mice [166]. The PTEN status in vitro has been shown to be a crucial determinant of glioma cell fate after ionizing radiation exposure; PTEN-mutant cells underwent premature senescence, while cancer cells expressing PTEN underwent apoptosis [167]. In addition, the inactivation of PTEN resulted in p53-mediated senescence and suppression of tumorigenesis in mice [94]. While these genes are mediators in the senescence response of cancer cells, they are not essential for senescence induction in cancer [86].

#### Changes in association with immune molecules and cells

**Fig. 4:**
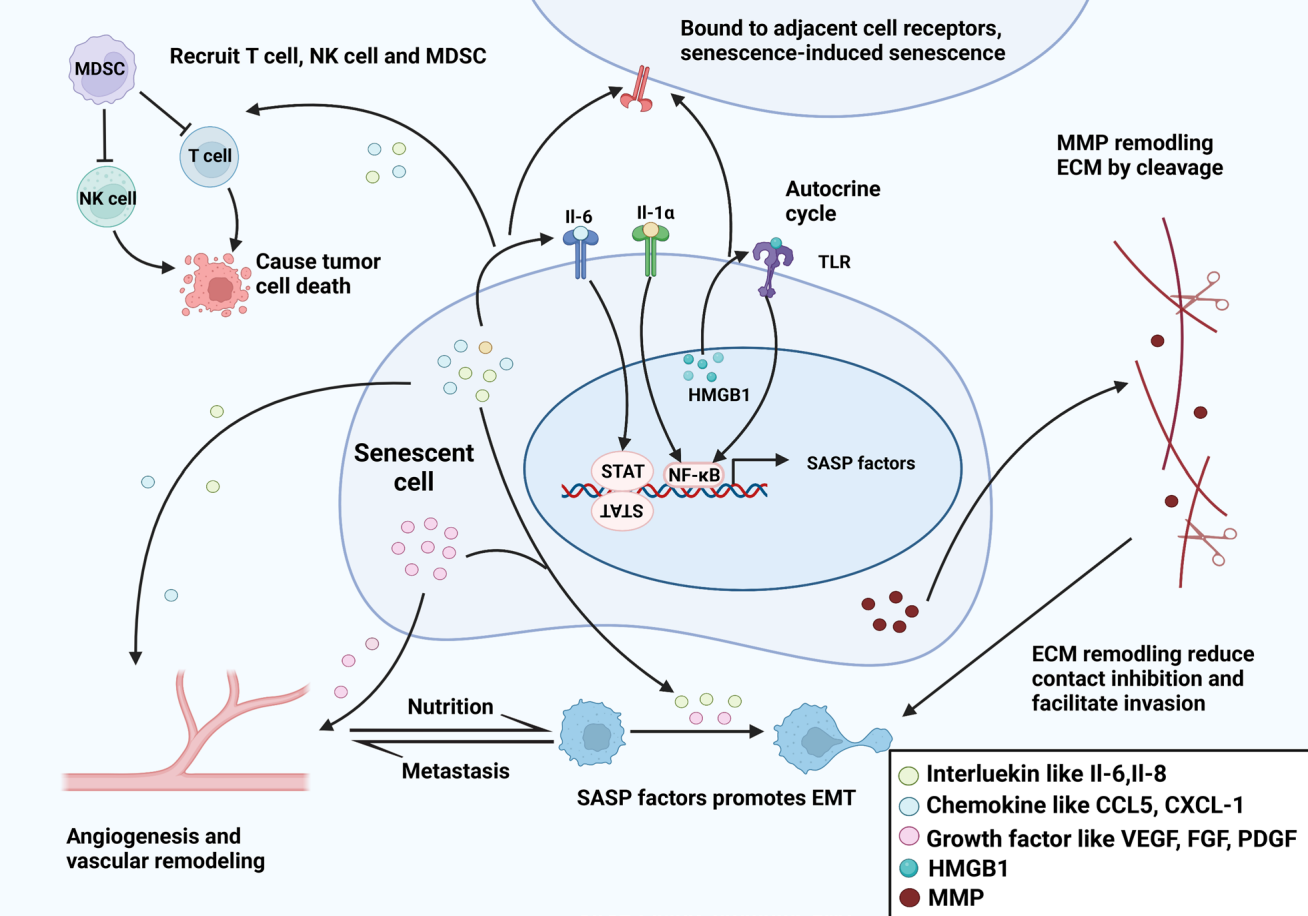
Components and potential effects of senescent cell in tumor. NF-κB signaling is activated in senescent cancer cells and elevates multiple production of IL-1α, IL-6, IL-8, CCL5, and growth factors like VEGF, FGF, PDGF, HMGB1, and MMP. To be detailed, IL-6, IL-8, CCL5, CXCL1, etc., help to recruit MDSCs to the TME to mediate protumorigenic effect of senescent cells, which block IL-1α signaling and antagonize the establishment of senescence in cancer cells. Simultaneously, MDSCs block immune surveillance by inhibiting CD8+ T cells and NK cells through IL-6 and CCL2, respectively, while IL-6 and IL-8 can recruit NK cells and T cells to reinforce immune surveillance. The ILs will spread senescence to surrounding cancer cells in a paracrine fashion, which further mediates tumor growth. The prominent SASP factors are involved in ECM processing and degradation, which can promote tumor cell proliferation and invasion. IL-6 secreted by senescent cancer cells or released from the ECM by MMPs recruits MDSCs, leading to an immunosuppressive TME. Moreover, the cleaved ECM components release growth factors such as VEGF, FGF, PDGF, and ILs that can promote tumor growth and EMT, promoting tumor metastasis. Additionally, MMPs also promote the release of many other cytokines and growth factors such as VEGF supporting tumorigenesis and chemokine CXCL1 to promote tumor growth. The SASP can stimulate blood vessel formation and vascular remodeling that contributes to tumor metastasis. Activated TLR4 promotes tumor progression in breast, prostate, and colon cancers and is associated with poor prognosis, but the antitumor activity is increased in skin cancers. TLR4 also recognizes HMGB1 and facilitates SASP phenotype formation. NF-κB, nuclear factor-κB; VEGF, vascular endothelial growth factor; FGF, fibroblast growth factor; PDGF, platelet-derived growth factor; MMP, matrix metalloproteinases; IL, interleukin; TME, tumor microenvironment; MDSCs, myeloid-derived suppressor cells; NK cell, natural killer cell; SASP, senescence-associated secretory phenotype; ECM, extracellular matrix

The SASP can affect the tumor microenvironment in many ways (Fig. 4). SASP components include immunoregulatory factors, including IL-6, IL-8, and MCP-2; growth factors, including HGF and IGFBP; and exfoliated cell survival factors, including ICAMs and UPAR [28]. As an autocrine proinflammatory factor, Il-1α binds to cell receptors to form a positive feedback mechanism mediated through the NF-κB pathway, which might maintain the SASP and the secretion of IL-1β, IL-6, and IL-8 [168, 169]. A previous study showed that IL-6 functioned through complex mechanisms. Under oncogenic stress, IL-6 is an autocrine factor that inhibits cell proliferation via cell cycle arrest. However, when acting as a paracrine factor, IL-6 promotes angiogenesis, which contributes to tumor progression [29]. Other SASP components, namely, chemokines, function in the tumor microenvironment. Multiple studies have confirmed that CCL5 inhibits the activation of Th1 cells and cytotoxic T cells and recruits MDSCs, T-regulatory cells (Tregs), and mesenchymal stem cells (MSCs), which reduce the killing ability of T cells and NK cells [170]. Moreover, CXCL1 induces adjacent cell senescence and immune escape through paracrine signaling [171]. These secreted immunoregulatory factors might lead to chronic inflammation and contribute to the transformation of an immunosuppressive microenvironment by regulating immune system cell infiltration [160].

From an overall perspective, aged individuals are often in a state of chronic inflammation. Obesity, changes in intestinal microbes, and tissue degradation exacerbate this chronic inflammation. Compared with those in young people, the serum levels of IL-1, IL-6, IL-8, and TNF-α in aged people are significantly increased [172]. This chronic inflammatory condition and the interrelated pathways usually exert an immunosuppressive effect and can increase the risk of cancer [22, 173]. Increased IL-6 and IL-8 levels can promote the EMT and tumor cell invasion [174]. The specific inflammatory environment also leads to the accumulation of Treg cells, Th2 cells, and activated B cells, increasing the secretion of IL-4, IL-6, IL-10, IL-33, and TGF-β, which are important growth-promoting factors [175].

In addition to molecular changes, changes in immune cell receptors, especially T-cell receptors, are important in the elderly tumor microenvironment. In contrast to highly expressed suppressing molecules in mice, the levels of PD-1 and TIM-3 in human T cells were not significantly changed, but CTLA-4 and LAG-3 were slightly elevated during senescence [176]. During aging, the proportion of CD8+ T cells that do not express CD28, a co-stimulatory molecule, increases, and this increase is accompanied by the upregulation of CD57 and killer inhibitor receptor (KIR) [177]. The immunoreceptor tyrosine‐based inhibitory motif (ITIM) domain protein (TIGIT), which is highly expressed in elderly CD8+ T cells, can exert an immunosuppressive effect on CD226 by competing with the ligand CD155 [176]. In addition, the expression and activity of CD38, a key molecule in NAD + depletion, are upregulated with advancing age. Activated CD38 may play an immunomodulatory role and has been considered a new immune checkpoint [178]. In addition to the aforementioned CD molecules, TLRs are involved in aging. The role played by TLR4 is tissue-specific. Activated TLR4 promotes tumor progression in breast, prostate, and colon cancers and is associated with poor prognosis but shows antitumor activity in skin cancers [179]. TLR4 recognizes HMGB1 and facilitates SASP acquisition [180]. Experiments have shown that the expression of TLR4 is elevated in aging mice [181]. This may indicate that TLRs are also involved in changes in immune cells in the aged tumor microenvironment.

#### Changes associated with cell growth

During the aging process, molecules and receptors of growth factors exhibit significant functions and tissue specificity in various ways. GDF 15, an important growth factor in the SASP, promotes epithelial cell proliferation, migration, and invasion through the MAPK and PI3K signaling pathways, thereby promoting tumor progression [182]. In addition, in a family of receptor protein tyrosine kinases, ErbB receptors directly or indirectly interact with classical downstream pathways such as MAPK, PI3K, and JAK [183]. The regulatory effects of ErbB on tumor cell proliferation, progression, and invasion have been widely studied in colorectal, breast, and lung cancers [184–186]. Among the ErbB family members, ErbB-1 (EGFR) binds to EFG, another SASP component, and promotes cell division [187, 188]. Moreover, clinical trials have revealed that aging is a risk factor for advanced lung cancer with EGFR mutant subtypes [189]. In vitro studies have also shown that p53 induces senescence by downregulating EGFR in multiple cell lines [190]. This finding is consistent with the opinion that aging prevents the growth of cells that are at risk of tumor transformation and thus inhibits tumorigenesis [191]. In addition, FGFR inhibitors have been reported to inhibit breast, gastric, and clonal cancers [192–194]. Ota et al. showed that FGFR promotes DNA-associated senescence, but loss of p53 and dysregulation of c-Myc reversed this effect and promote tumor transformation. However, in the same study, activation of FGFR can downregulate the expression of c-Myc, which may have suppressed tumor formation [195]. Additionally, FGFR may also act as a negative regulator of mesenchymal stem cell senescence. Reduced expression of FGFR has been revealed in a variety of aging tissues [196]. By interacting with RACK1, FGFR promotes the degradation of p53 in lung squamous cell carcinoma, ultimately inhibiting tumor cell senescence [197]. By binding to FGFR, sulfated heparin prevents premature replicative senescence and ultimately prohibits p53 expression. The indirect depletion of surface sulfated heparin leads to cellular senescence in tumors. Therefore, Jung et al. speculated that FGFR initiates new tumor defense mechanisms by regulating premature senescence [198]. The evidence illustrates a regulatory role and increased complexity of growth factors in aging and tumorigenesis.

As an upstream and downstream molecule regulated in vivo, GH regulates the secretion of IGF-1, which constitutes the GH/IGF-1 axis. As the corresponding hormone receptors, GHR and IGFR play important roles in cell senescence and tumor formation. Most tumor cells express GH, which may indicate that the autocrine function of GH on tumor cells activates GHR more than GH secreted by the pituitary gland, thereby driving cancer progression [199]. Correspondingly, in patients lacking GHR (Laron syndrome), the concentration of IGF in the patient's serum is significantly reduced, but these patients are free from aging-related disorders and rarely develop tumors [200]. Similarly, overexpression of IGF-1R has been observed in various tumors, such as those in thyroid cancer and breast cancer [201]. Additionally, these receptors may all be engaged in aging. Strous found that GH knockout significantly extended the lifespan of mice and delayed immune system-related aging. In addition, they observed the downregulation of the GH/IGF-1 axis activity in elderly individuals [199]. Similarly, it has been confirmed that the lifespan of mice was significantly prolonged after heterozygous IGF-1R knockout [202]. A similar conclusion suggested that in individuals older than 100 years, the activity of IGF-1 is reduced, which may be mediated by IGF-1R mutation, and these individuals exhibit profound anti-inflammatory characteristics. These two changes both depend on p53, which reduces the risk of tumor development [203, 204]. The activation of IGF-1R leads to the activation of PI3K, Ras-Raf, JAK/STAT3, and other pathways, ultimately upregulating p21, which promotes cell proliferation, survival, migration, and adaptation to hypoxia and inhibits autophagy, apoptosis, and anoikis [205]. Therefore, we speculate that IGFR may be involved in the forkhead pathway in cellular senescence and tumor formation. The ultimate fate of a cell may depend on which of the two states prevails. In formal theory, in the early phase, aging inhibits tumor formation by preventing the growth of cells [191]. In this process, cell surface receptors activate a variety of signal transduction pathways and engage in crosstalk with each other, ultimately affecting the activity of p53 and other key proteins. Intracellular receptors can also sense various changes in senescent cells and ultimately participate in their regulation. All of these factors might reflect constant competition between aging and tumor cells.

### Impact of senescence on the TME

#### The effects of the ECM

The tumor microenvironment matrix constitutes endothelial cells, fibroblasts, pericytes, adipocytes, immune cells, and the ECM, which is composed of collagen, fibronectin, laminin, and elastin [3]. Matrix fibroblasts inhibit tumor cell proliferation [206]. The different cross-linking and arrangements of ECM proteins change the physical features of the ECM, such as its stiffness and mechanical force [207]. Moreover, through focally assembling integrin adhesion complexes, cells can sense changes in the ECM and regulate the cell cycle and energy metabolism via the FAK/Src, ILK-PINCH-parvin-kindlin, and α-actinin-zyxin-VASP signaling pathways [208]. This molecular and supramolecular heterogeneity affects the infiltration and migration of cancer cells and angiogenesis [207]. Moreover, the ECM contains growth factors, including IGFS, FGFs, TGF-β, and HGF [209]. These stimulating factors can induce adipocytes, mesenchymal stem cells, pericytes, and other cells to transform cancer-associated fibroblasts (CAFs). Similarly, miRNA-21 induces CAF formation via its inhibition of the Smad7 pathway. These CAFs induce ECM remodeling by secreting matrix metalloproteinase (MMP) [210, 211]. On the basis of the product levels of unique collagen and other ECM molecule genes, such as *COL10A1* and *COL4A1*, a previous study showed that CAFs can be classified into multiple subtypes. These multiple CAF subtypes increase the complexity and heterogeneity of the tumor microenvironment [212].

Many time-related mutations accelerate normal stromal cells, which are transformed into CAFs, contributing to immune cell regulation, angiogenesis, and ECM remodeling. The promotion of CAF activation may be a result of the impaired function of p53/p21 and CLS/RBP-Jκ, a transcription factor in dermal fibroblasts, which functionally and physically interacts with p53 in the Notch signaling pathway [213]. Activated CAFs upregulate the expression of VCAM-1 in adjacent tumor cells and promote the adhesion of monocytes. Several studies have shown that activated M2 macrophages might, in turn, promote the formation of CAFs, thus participating in crosstalk with malignancy factors in pancreatic cancer and neuroblastoma [214, 215]. CAFs also inhibit NK cells activate receptors on the surface of cells and killer particles [216] and recruit normal DCs to form IDO-producing regulatory dendritic cells [217]. Moreover, CAF cells stimulate miR21/Toll-like receptors through lactate to promote CD4+ T-cell polarization from Th2 to Th1 cells and maintain Treg cells [218–220]. The number of CD8+ T cells is reduced by CAF-upregulated immune checkpoint molecules such as PD-1, which facilitates tumor cell immune escape [221]. Senescent fibroblasts express the nonclassical MHC molecule HLA-E, which inhibits the immune response by interacting with NKG2A on the surface of NK cells and CD8+ cells [222].

In the aging tumor microenvironment, change in the ECM is an important factor. As other secreted SASP factors, MMP-1, MMP-3, MMP-10, and other MMP levels are elevated in the aging matrix, which contributes to ECM remodeling [3]. Through this increased MMP expression, senescent cells show reduced contact inhibition, facilitating cancer growth. Under the action of MMP, the collagen in the ECM undergoes fibrosis, accompanied by the destruction and reorganization of the elastin structure, composition alteration of laminin, and decreased hydration capacity of hyaluronic acid, eventually resulting in the loss in ECM mass and moisture, increased fibrosis, and tissue dysfunction [223]. A study demonstrated that fibronectin inhibits tumor cell proliferation in the tumor microenvironment but plays the opposite role in the normal stroma. In the aging ECM, the expression of fibronectin is upregulated; however, due to hypoxia, mutations, nutritional deficiency, viral infections, and other adverse factors, structures often undergo misalignment [224]. The decline in heart function in old age enhances the chances of ECM [225]. In addition, the decline in NAD+ levels in senescent cells leads to increased stability of HIF-1α, which induces a pseudohypoxic state [226]. Activation of HIF-1α can mediate cancer cell invasion through the action of fibronectin [227]. Hypoxia can also change the stiffness of the ECM mediated through LOX, leading to immune cell infiltration and tumor cell migration [228–230].

#### Angiogenesis

In young individuals, angiogenesis vitally contributes to tumor progression. Previous studies have shown that solid tumors need enough blood to grow. When solid tumors are larger than 2 mm in diameter, new blood vessels must be formed to maintain the blood supply; without new vasculature, the tumor undergoes necrosis due to hypoxia [231]. In addition, angiogenesis is related to tumor cell invasion, immune cell infiltration, and chronic systemic inflammation.

In normal tumors, angiogenesis can be initiated in many ways, including vasculogenesis, sprouting angiogenesis, and vasculogenic mimicry (VM) [232]. Vasculogenesis refers to endothelial progenitor cells (EPCs) differentiating into endothelial cells and de novo formation of a primary blood vessel network. Sprouting angiogenesis refers to the proliferation and migration of existing vascular endothelial cells, which generate new capillaries, which is the process in embryonic development, after birth and during tumor progression [233]. Moreover, in the tumor microenvironment, a variety of angiogenic factors, including VEGF, FGF, and PDGF, promote angiogenesis [234]. In contrast to the two aforementioned methods, VM does not depend on endothelial cells [235]. Many studies have reported that VM is involved in a variety of tumors [236]. In the VM process, tumor cells are arranged into tubes and covered by glycoproteins to form blood vessels [232]. There is evidence to suggest that VM may be the blood supply source in early tumor progression and that these vessels are gradually replaced via vasculogenesis and sprouting angiogenesis in a later stage [237]. Regardless of the mechanism, angiogenesis is closely related to hypoxia. Under hypoxic conditions, the content of HIF-1α increases. Activated HIF upregulates the expression of VEGF, TGF, and other angiogenic factors; IGF, c-Myc, and other proteins associated with cell survival and proliferation; and GLUT1, GLUT3, and other proteins that change cell metabolism and enhance adaptability [238]. Moreover, hypoxic conditions can accelerate the EMT of tumor cells and the secretion of MMP, which leads to an incomplete vascular matrix that enables cells to migrate, thereby increasing the aggressiveness of tumor cells [239]. Circulating tumor cells (CTCs) originating from a primary focal point may die rapidly after entering the blood due to the shear force of the blood flow and anoikis [240, 241]. However, CTCs can interact with blood platelets, macrophages, lymphocytes, and other cells to prolong survival and immune escape [242]. In addition, a study demonstrated that the microvasculature may be a premetastatic niche formed by tumor cells before they enter the circulatory system [243].

In the aging tumor microenvironment, the change in angiogenesis mechanisms is diverse. A study showed that SRPX expression is increased in senescent cells and decreased in tumor cells, and SRPX itself promotes angiogenesis through the FAK pathway [244]. Thus, cells acquire the SASP during senescence. Among SASP factors, VEGF, PDGFA/B, FGF, and other secreted angiogenic factors and CCL5, CXCL-1, Il-6, and other secreted proinflammatory factors may promote vascular remodeling [37]. Changes in miRNA expression during cell senescence may promote tumor metastasis. Mir-21 can inhibit the regeneration of endothelial cells and promote angiogenesis in vitro and in vivo, and it is secreted by CAFs in exosomes [210, 245]. In addition, previous reports have noted that exosomes carrying miRNA can regulate the microenvironment of a metastatic site, thereby promoting tumor colonization [211]. In addition, HDAC, which functions as part of the SASP, also contributes to vascular endothelial formation. Many studies have shown that HDAC6, HDAC7, and HDAC9 promote angiogenesis by inducing endothelial cell migration, while HDAC5 exerts an antiangiogenic effect in endothelial cells [99, 246–248].

Moreover, angiogenesis in the tumor microenvironment can be affected by changes in the aged physiological state. Previous studies have shown that with advancing age, the number of small arteries in certain tissues decreases, which decreases blood flow and in turn leads to the downregulation of Notch in endothelial cells [249]. As mentioned above, MMP9 is highly expressed in the heart during aging, causing collagen deposition and cross-linking [250]. ECM deposition can cause cardiac insufficiency, reduce angiogenesis and further reduce the blood supply to the whole body [225]. These mechanisms together reduce blood flow into the tumor microenvironment, causing hypoxic and nutrient-deficient conditions, which activate HIF and other signaling pathways. Moreover, studies have shown that molecules including Il-4 and CD163 secreted by M2-polarized macrophages can induce pathological angiogenesis, which may increase the blood supply to the tumor microenvironment [251]. In addition, CTCs can be cloaked with platelets, reducing the effect of the shearing force of the blood. Platelets can also support CTC adhesion to the vasculature and facilitate CTC immune escape [242, 252, 253]. In aging bone marrow, macrophages promote an increase in platelet hematopoietic stem cells through the action of Il-1, which ultimately increases the number of circulating platelets in mice [254]. These mechanisms together maintain the material supply to an aged tumor microenvironment.

#### Stromal population

**Fig. 5:**
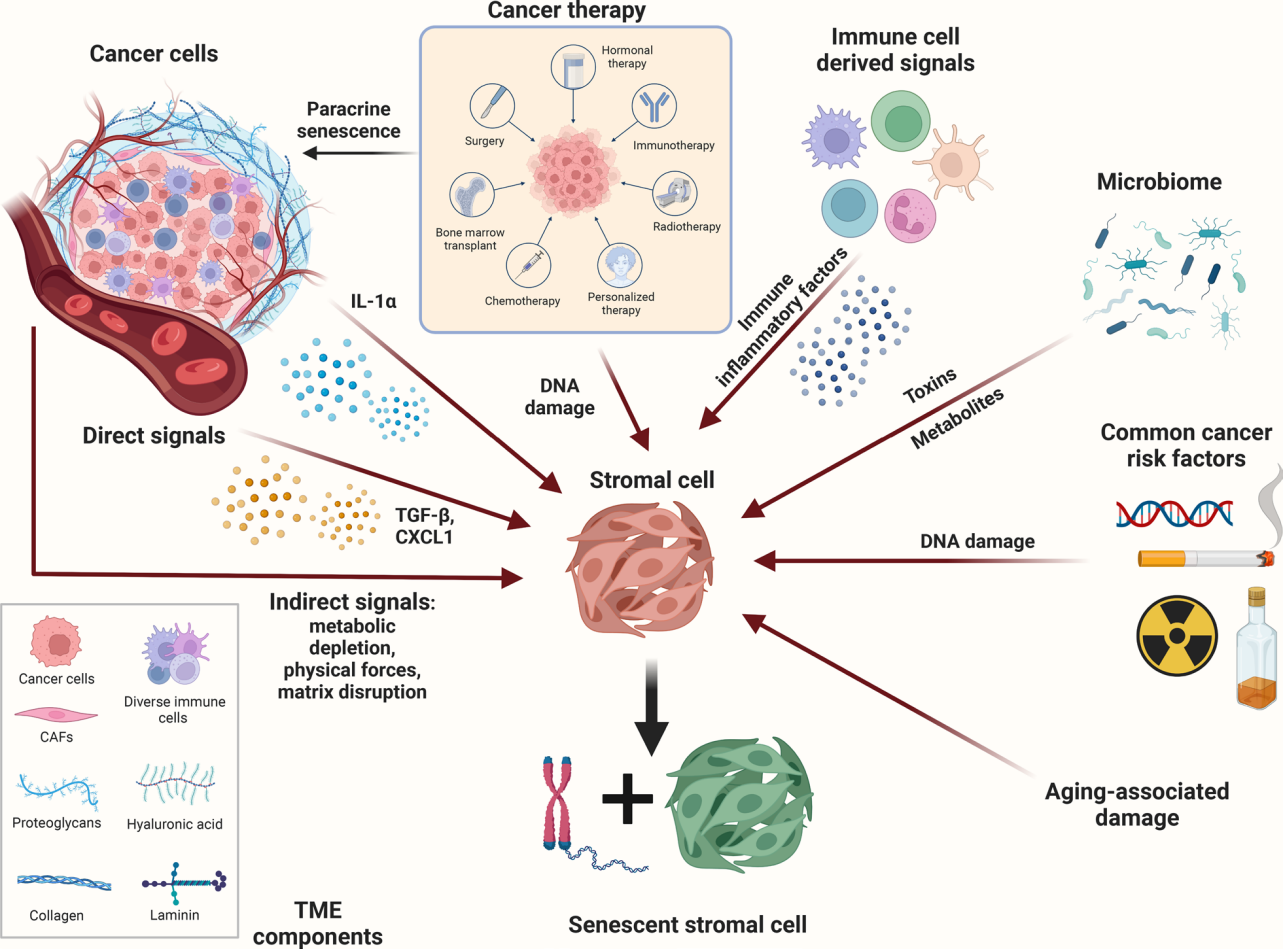
Potential risk factors for stromal cell senescence. Senescence can be induced in stromal cells with different signals from different avenues. Cancer cells can release cytokines and growth factors (IL-1α, TGF-β, CXCL1) that induce stromal cell senescence directly. Cancer cells can alternate TME via regulating metabolism, environmental stress, physical forces, and matrix disruption to indirectly induce stromal cell senescence. Different types of cancer therapy (i.e., chemotherapy, radiotherapy, immunotherapy, and personalized therapy) may have the possibility to induce paracrine senescence of cancer cell senescence and induce stromal cell senescence via DNA damage signal. Immune cell may emit diverse immune inflammatory factors to promote stromal cell senescence. Microbiota in the gut or TME can also cause stromal senescence by generating toxins and metabolites. The common cancer risk factors including alcohol, smoking, radiation, and genetic disease are associated with DNA damage signal to induce stromal senescence. Furthermore, other age-associated damage signals may arouse stromal senescence. TME, tumor microenvironment; CAFs, cancer-associated fibroblast (Mainly from 10.1016/j.trecan.2022.09.002)

The stromal TME within tissue is made up of various components, including fibroblasts, endothelial cells, pericytes, adipocytes, the ECM, and immune cells, and plays a major role in TME homeostasis. Fibroblasts are the most common stromal component; these cells are required for the synthesis of collagen and for the structural integrity of connective tissue and play key roles in wound healing and inflammation [255]. Their predominant mode in regulating many of these processes is through the secretion of soluble factors, including cytokines, chemokines, growth factors, enzymes, and structural components of the ECM [23, 38]. Given the complexity of different tissues, the TME plays a specific role in the regulation of the soluble factors secreted by fibroblasts along with their migratory and proliferative characteristics. The fibroblast renewal rate, defined as the sum of the total number and proliferative capacity of fibroblasts, greatly varies among different tissues, with factors such as local temperature, vascularization, mechanical stress, and hormonal responses contributing to the renewal rate [256]. Changes in fibroblasts during aging are likely to be different between organ sites and often involve senescence. Senescence is a classic example of antagonistic pleiotropy, and the accumulation of senescent cells is a key pathological feature associated with aging [257–260]. Cellular senescence is linked to many of the cellular processes of aging and can be a direct result of responses to intrinsic or extrinsic oncogenic stimuli; notably, many forms of senescence are not aging-related (e.g., oncogene-, replication-, stress- and therapy-induced) [261–263] (Fig. 5).

There are still some disputes on how the accumulation of senescent cells occurs in elderly individuals. It should be hypothesized that, as we age, a reduction in immune function decreases the recognition and clearance of these growth-arrested cells, which eventually results in their accumulation [264]. Typically, the SASP is believed to be made up of approximately 75 secreted factors, including granulocyte–macrophage colony-stimulating factor (GM-CSF), IL-6, IL-8, and IL-10 [38, 260]. However, many of these secreted factors were identified in studies using oncogene-induced senescence models and may not necessarily reflect true age-induced senescence. While the mechanisms underlying age-related SASP transformation are still under investigation, many genetically engineered mouse models (GEMMs) have been key in determining their pathological and homeostatic role. *p16*^*INK4A*^ activation seems to be one of the important contributors toward senescence induction in cells [265], yet the contributions to age-related accumulation of senescent cells need more description. To be consistent, when modeled in vivo, its contribution toward senescent cell accumulation is described as more of a “molecular” form of aging as opposed to a “chronological” form [265]. The GEMM demonstrates that a dramatic accumulation of *p16*^*INK4A*^-expressing cells occurs across various tissues throughout the aging process and characterizes the pathological changes associated with the age-induced SASP in peritoneal macrophages, illustrating the potential for other stromal components. Fibroblasts contribute to many SASP-related pathologies, and studies have shown that tumor-associated fibroblasts undergo chromatin remodeling via histone deacetylase (HDAC) modulation to achieve a SASP irrespective of DNA damage [99, 266]. Recently, it was shown that LINE-1 retrotransposable elements are derepressed at the transcriptional level to elicit a type I IFN response, which contributes to the maintenance of a SASP [59]. These findings further support the conclusion that dynamic changes within an aged TME play a key role in reprogramming cells toward a SASP.

#### Other TME components

Other senescent cell populations, such as endothelial cells, epithelial cells, immune cells, stem cells, and even certain tumor cells, play clear roles in modulating the TME by acquiring the SASP [21, 267]. Many examples have shown that senescent cell populations can contextually produce protumorigenic or antitumorigenic effects; however, direct age-related evidence for these effects within these cell types remains limited. A recent study based on a xenograft model with human BPLER triple-negative breast cancer cells in nude mice found that tumors showed delayed onset, slower growth kinetics, and reduced metastasis in aged mice (> 10 months old) than in young mice (8–10 weeks old). Furthermore, a subset of tumor-infiltrating hematopoietic cells in young mice showed upregulated CSF1 receptor (CSF1R) expression and secreted the growth factor granulin to induce robust tumor growth and metastasis. Importantly, bone marrow-derived cells from young mice and transplanted into aged mice were sufficient to activate the tumor-supportive TME and induce tumor progression.

Cellular senescence has been confirmed to be a key contributor to inflammaging in many age-related malignancies [268, 269]. SASP acquisition in stromal cell populations results in the persistently increased secretion of multiple inflammatory cytokines that maintain low adaptive immune response levels. Other age-related changes to the gut microbiota, obesity, and tissue degradation also appear to drive the inflammaging response [22]. In total, these age-related processes appear to drive chronic inflammation by increasing systemic levels of IL-1, IL-6, IL-1α, IL-1β, IL-33, GM-CSF, IFN-γ, TNF, and C-reactive protein (CRP). All these factors contribute to multiple morbidities and mortalities in elderly individuals [22, 270]. Similar effects have also been observed with increased infiltration of immunosuppressive Treg cell populations in chronic inflammatory mouse models, mimicking the chronic inflammation that often precedes and may lead to certain malignancies such as melanoma and colorectal cancer [271]. Treg cells play a key role in maintaining tolerance to self-antigens and suppressing the induction and proliferation of effector T cells (such as CD4+ and CD8+ T cells) via the secretion of cytokines and enzymes [272]. Another study induced chronic, tumor-promoting allergic contact dermatitis (ACD) in 6- to 8-week-old mice by treating them with 1-fluoro-2,4-dinitrobenzene (DNFB) and found that IL-33 expression was key in inducing the transition from acute, tumor-suppressing inflammation to chronic inflammation [271]. The number of Treg cells was significantly reduced in DNFB-treated IL-33-knockout mice, and knocking out IL-33R in Treg cells significantly reduced ACD-induced skin carcinogenesis. Interestingly, in colitis-induced colorectal cancer, the IL-33-Treg cell axis was identified as a key driver of carcinogenesis. However, more evidence on the age-related effects of the IL-33-Treg cell axis in tumorigenesis is needed [270, 273, 274].

Several microRNAs (miRNAs) termed identified in association with many human malignancies are called “inflamma-miRs” [275]. Age-related increases in miR-19b, miR-21, miR-126, and miR-146a appear to drive the progression of many types of cancer, and possibly other diseases, through inflammaging [276]. miR-21 has been shown to be overexpressed in many malignancies and to reduce the expression of the potent anti-inflammatory factors IL-10 and TGF-β [277], but when binding to Toll-like receptor 8 (TLR8), it induces the secretion of the inflammaging cytokines IL-6 and TNF [278]. Immunosenescence is another contributor to many age-related pathologies, including cancer. Immunosenescence is defined as the age-related dysregulation of the immune system, whereby subpopulations of effector immune cells and overall immune function decline. This process is a result of multiple factors, including thymic atrophy [279], a decrease in the number of naive T cells [280], a reduction in memory T-cell function [281], and decreased recognition of diverse antigens by T cells [282, 283]. Along with T-cell dysfunction, NK cells, macrophages, and dendritic cells, all of which play early roles in tumor-immune recognition and suppression, appear to undergo phenotypic decreases in cytotoxic activity as humans age [284–287]. The key involvement of these processes in cancer progression has been identified in a squamous cell carcinoma (SCC) model of aging [288]. In GEMM models of conditionally expressed mutant HRAS in keratinocytes, aged mice (18–22 months) developed SCC more quickly than young mice (2–4 months). Molecular analyses of the immune system of the aged mice revealed a shift toward a protumorigenic T helper 2 cell anti-inflammatory response, as well as increased expression of inhibitory programmed cell death ligand 1 (PD-L1) and senescence-associated β-galactosidase on effector immune cells in the dermis.

**Fig. 6:**
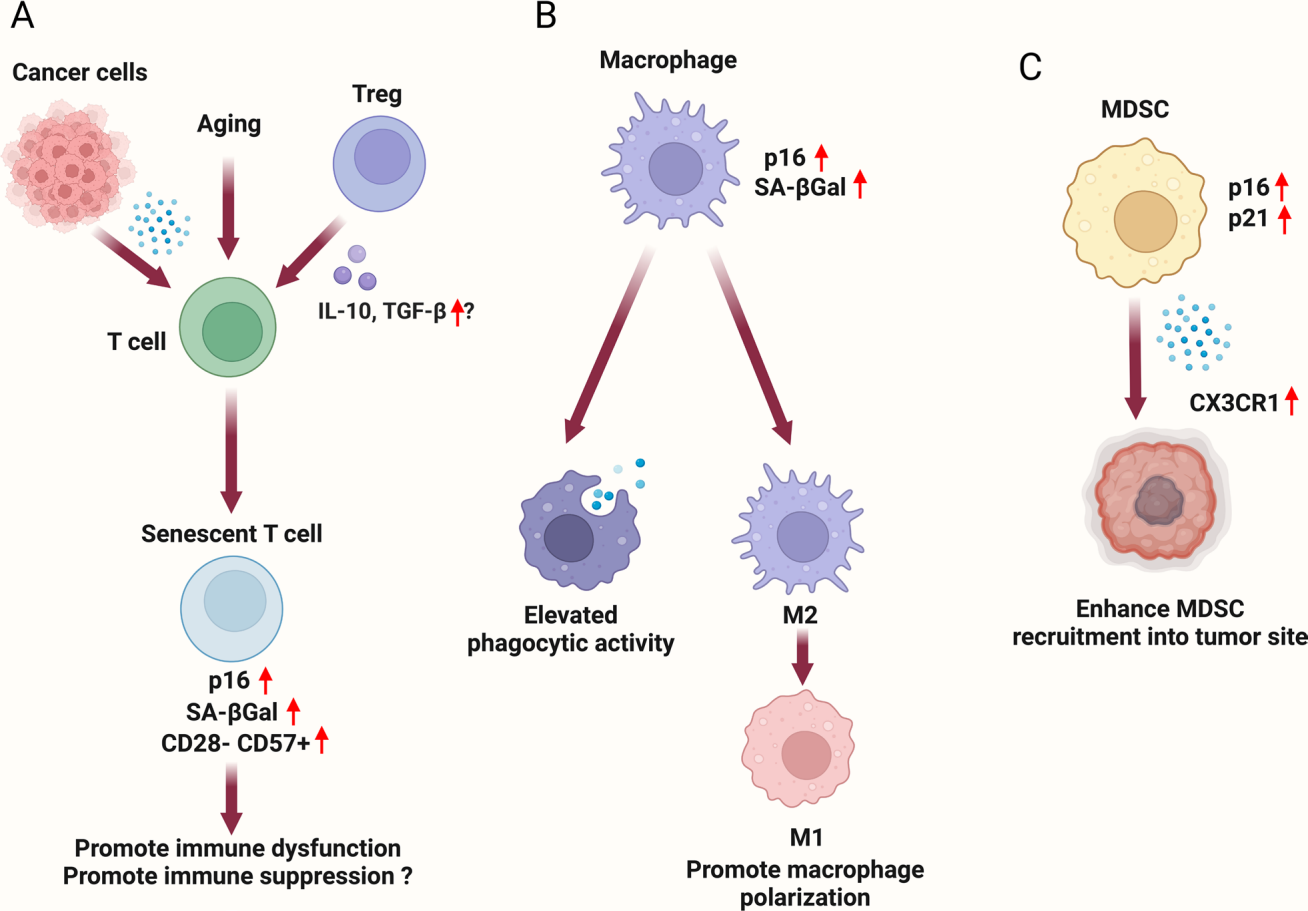
Biological function and effect of senescent immune cells. **A** T-cell senescence may be activated by soluble factors secreted by cancer cells, Tregs, and occur during the aging process. The senescence markers such as p16, SA-βGal are elevated, co-stimulatory receptors (CD27, CD28) are decreased and CD57 is increased. Senescent T cells may have poor immune function and reveal immune suppressive TME. **B** p16 and SA-βGal highly expressed, senescent macrophages may enhance phagocytic activity or increase macrophage polarization by transferring M2 into M1. **C** In senescent MDSCs, p16 and p21 are highly expressed and upregulate the expression of the chemokine receptor CX3CR1, which mediates the recruitment of MDSCs to tumor site. Tregs, regulatory T cells, MDSCs, myeloid-derived suppressor cells (Mainly from 10.1016/j.trecan.2022.09.002)

Age-induced immunosenescence is largely mediated by effector T cells and other immune cell types required for antitumor immunity (Fig. 6). Changes in these cells have been hypothesized to induce a shift toward the activation and infiltration of more immunosuppressive cell populations in elderly individuals, which is important to their increased predisposition to cancer cell invasion and metastasis [289]. The number of M2 tumor-associated macrophages (TAMs), which are negatively associated with tumor immunity, is significantly higher in the spleen and bone marrow of aged mice (> 24–28 months old) [290]. Additionally, stimulation of macrophages isolated and cultured from aged mice in mesothelioma or lung carcinoma cell-derived culture supernatants increased the levels of the M2-derived immunosuppressive cytokine IL-4. Stimulation of M2 TAMs toward switch to an M1 proinflammatory phenotype by treating aged mice with a combination consisting of an IL-2 agonist and anti-CD40 therapy reduced immunosuppressive IL-4 and IL-10 expression and inhibited tumor growth [291]. While the binary M1/M2 classification of macrophages has been hotly debated, a large amount of evidence suggests that M2-like immunosuppressive macrophages promote tumor progression in an aging context [291]. TAMs have also been demonstrated to play key roles in establishing a premetastatic niche in the liver by secreting CXCL1 and inducing the recruitment of MDSCs, which are necessary for the efficient formation of colorectal cancer liver metastases [292]. *p16*^*Ink4a*^ and *p21*^*Waf1*/*Cip1*^ could suppress tumor progression by inducing cellular senescence, the deletion of *p16*^*Ink4*^ and *p21*^*Waf1*/*Cip1*^ reduces CX3CR1 expression and inhibits monocytic-MDSCs (Mo-MDSCs) accumulation in tumors expressing CX3CR1, hence suppresses the tumor proliferation in mice model. The regulation of Mo-MDSCs is a valuable strategy to inhibit tumor progression [293]. More direct evidence on the systematic effects of M2 TAMs is warranted in the future.

Neutrophils undergo immunosenescence throughout aging, and they have been confirmed to infiltrate injured tissue in elderly people [294]. Studies have shown that neutrophils in aged patients and mice produce more anti-inflammatory cytokines than their younger counterparts [295]. Neutrophils are well-characterized regulators that mediate tumor progression through proinflammatory effects in tumor models of young mice; however, subpopulations of anti-inflammatory “N2 tumor-associated neutrophils (TANs)” have recently been implicated in many kinds of cancers [296, 297]. Studies conducted with young mice revealed that the number of immunosuppressive N2 TANs is systematically increased in aged patients and that they exhibit a function similar to that of MDSCs [298]. Nevertheless, direct age-related investigations into N2 TAN involvement in the TME should be performed.

Many studies suggest a dramatic increase in Treg cell numbers and function in age-related pathologies and in organs such as the lymph nodes and spleen [299–302]; however, other reports showed no change in Treg cell numbers or function or their reduced contribution to other aged tissues and cancers [303–305], suggesting that Treg cells may play a context-specific role in different TMEs and cancer environments. Furthermore, Treg cell recruitment also appears to be a significant factor in the establishment of the premetastatic niche in many cancer types [306]. Nevertheless, whether the age-related increases in the number of Treg cells that is observed systematically in certain mouse models are directly linked with increases in age-related metastasis remains unclear. MDSCs exhibit a consistent increase in human blood during aging [307] and in the bone marrow and lymphoid organs of 17- to 19-month-old mice [308, 309]. Regression within young mice harboring breast cancer correlated with significant effector T-cell infiltration, whereas aged mice showed significantly increased numbers of MDSCs in the TME. Importantly, MDSCs are among the immune cell types most closely associated with the formation of the premetastatic niche in cancer [310]. Stromal senescence significantly increased the numbers of immunosuppressive MDSCs and Treg cells adjacent to senescent populations in healthy mice, primarily via the secretion of IL-6 [78]. These studies additionally suggest that the accumulation of senescent stromal cells is sufficient to establish a tumor-permissive chronic inflammatory TME that allows tumors to grow and progress unabated by the immune system. Age-related increases in the numbers of systemic MDSCs and acceleration of other age-related processes, such as inflammaging and ECM modulation, may directly link MDSCs, Treg cells, and other immunosuppressive cell subpopulations with age-related cancer predisposition and premetastatic niche formation. Details on the roles played by immunosuppressive immune cell types in these processes are needed. In prostate cancer, effector T cells and proinflammatory cytokines appear to contribute to increases in tumor growth. Prostate fibroblasts cultured from young (< 55 years old) and aged (> 65 years old) healthy individuals showed that the aged fibroblasts secrete a greater number of cytokines and interleukins, which promote the growth of epithelial cells and may affect the function of immune cells [311]. Another study on prostate cancer showed that CD3+, CD4+, and CD8+ T-cell infiltration exerts a protumorigenic effect and is associated with tumor growth [312]. These findings suggest that effector versus immunosuppressive cell infiltration in the TME with advancing age depends on the context and is related to tumor growth and premetastatic niche formation.

## Aging and potential response to therapy

The treatment for cancer in elderly patients is still challenging because age-related health conditions often leave clinicians in a dilemma, as it is frequently unclear whether potentially beneficial therapies can be safely administered at standard dosages and will improve the prognosis or whether potential side effects will likely affect the patient’s quality of life (QoL). Approximately 50% of all cancers are diagnosed in patients over 65 years old, and this percentage may increase to 70% as life expectancy continues to increase, but survival data from clinical trials for patients above this age are relatively rare [313]. In fact, 40% of patients enrolled in cancer trials are over 65 and fewer than 10% are older than 75 years; therefore, more evidence is required to facilitate clinical management for elderly cancer patients. Recent advances in understanding the mechanisms of senescence, the aged TME, and responses to therapy will yield crucial knowledge to allow more efficient targeting and less-intensive treatment of different cancer types in elderly patients. In the following section, we provide descriptions of clinical therapy for patients presenting with both cellular senescence and cancer.

### Chemotherapy

Chemotherapy is a nonspecific, aggressive treatment that results in the targeting of fast-growing malignant cells; however, chemotherapy causes many side effects associated that can often be life-threatening in elderly individuals. A recent study showed that chemotherapy-induced senescent fibroblasts within mice caused a consistent inflammaging response, and the elimination of these fibroblasts significantly decreased both short-term and long-term side effects of chemotherapy-induced cytotoxicity, decreased the possibility of cancer recurrence and reduced the extent of cancer metastasis [88]. Many similar studies have suggested that chemotherapy may be initially beneficial; however, in many cases, it may later contribute to accelerated aging of the TME and increase residual disease in patients [314]. Chemotherapy can also induce off-target effects that include stem cell pluripotency decline and bone marrow exhaustion. Studies showed that MSCs in 16-month-old mice were much more sensitive to doxorubicin treatment than those in 1-month-old and 8-month-old young mice [315]. Patients can undergo stem cell transplantation while receiving high-dose chemotherapy regimens to prevent off-target effects; however, stem cell transplantation may induce toxicity that exceeds the safety threshold in many elderly individuals, which limits the broad use of this strategy [315]. In a cohort of patients with different types of cancers, including myeloma, lymphoma, and leukemia and who underwent hematopoietic stem cell transplantation, the expression of *p16*^*INK4A*^ was significantly increased in effector T cells. Further analysis of gene expression in effector T cells from these cancer patients showed clear signs of immunosenescence and T-cell aging [316]. The off-target effect associated with stem cell transplantation and chemotherapy can cause damage to the thymus by accelerating thymic aging [316]. Overall, there may be a potential clinical benefit in targeting chemotherapy-induced acceleration of age-related tumorigenic events to decrease cytotoxicity and increase survival. There are many cases where chemotherapy in elderly patients is tolerated well and prolongs survival irrespective of the location of the cancer [317]. In any case, the key consideration to determine proper chemotherapeutic regimens in the elderly should be whether the benefits of treatment outweigh the side effects. Further research is warranted to provide more insights into the cytotoxic effects at the molecular level and the degree of organ function decline in older patients.

### Targeted therapy

Targeted therapy has been regarded as one of the standard personalized approaches to target cancer cells or the TME for specifically inhibiting tumor development or progression. However, drug resistance and adverse events are always major concerns for this type of treatment [317, 318]. Targeted therapy often induces fewer off-target effects than chemotherapy or radiotherapy, as cancer cells targeted for therapy appear to undergo more intrinsic changes based on genetic mutations, epigenetic alterations, and genomic instability to induce off-target effects. A recent study illustrated that healthy aged dermal fibroblasts facilitated increased resistance to targeted BRAF therapy in allogeneic mouse models of melanoma by secreting SFRP2 into the TME [319]. Furthermore, using recombinant SFRP2 to treat young mice led to increased resistance in a formerly sensitive mouse model [319]. Studies focusing on melanoma have shown that B-cell infiltration in the TME results in the secretion of insulin-like growth factor 1 (IGF1), which promotes drug resistance to BRAF and MEK inhibitors [320]. Another recent study reported that immunosuppressive age-associated B-cell counts are significantly increased in aged mice (> 24 months old) but that the levels of other B-cell subtypes were reduced [320, 321]. The number of MDSCs, as previously described, increases with age, and these cells appear to induce resistance to antiangiogenic therapies as well as other targeted therapies used in the treatment of multiple myeloma [322], prostate cancer [323], liver cancer [324] and melanoma [325]; however, a direct link between MDSC drug resistance and age has not been investigated in these models. Notably, combination therapy with drugs targeting treatment resistance-promoting components in the stroma and ECM of the TME may increase the persistence of targeted therapy effects [326–328]. Given the complex mechanism by which protumorigenic effects are induced with age, research on the aging TME, potential premetastatic niches, and their contributions to targeted therapy resistance is definitely needed.

### Immunotherapy

Immunotherapy has been established to modulate the immune TME and thus target and eliminate tumor cells. The efficacy of immunotherapy is considerable; however, not all cancer patients receive clinical benefits from immunotherapy [329]. Therefore, investigators need to identify more-robust biomarkers for this kind of treatment. Marked changes in immune profiles and function are found in aging humans, and therapy targeting the immune system within the elderly has shown considerable clinical implications. However, few studies have incorporated elderly individuals into trials to evaluate the efficacy of immunotherapies in older cancer models. The most widely used immunotherapies that have proven clinical efficacy across a wide range of cancers are immune checkpoint inhibitors (ICIs) targeting programmed cell death 1 (PD-1), PD-L1, cytotoxic T lymphocyte-associated antigen 4 (CTLA-4) and lymphocyte activation gene-3 (LAG-3). Many cancer cells and other stromal components upregulate cell signaling through immune checkpoint pathways to evade antitumor immune responses. Interestingly, PD-1 surface expression has been suggested to contribute to the age-dependent functional decline of effector memory T cells [330]. PD-1 levels are increased on T cells with age, and anti-PD-1 therapy increases T-cell function, especially in aged mouse models [331, 332]. Rapamycin, an mTOR inhibitor, has been shown to reduce age-related increases in PD-1 levels, suggesting a role of these inhibitors in increasing tumor immunity in aged tissues [332]. A marked increase in PD-L1 expression in CD8+ effector T cells of aged mice compared with young mice has been observed, and anti-PD-L1 immunotherapy reduced cell proliferation in vitro and antitumor immunity in aged hosts compared with the effect in young mouse lymphoma models [333]. In another study, PD-L1 and indoleamine 2,3-dioxygenase 1 (IDO1) levels were increased during aging in the brains of healthy human adults, while the number of circulating Treg cells increased and that of CD8+ T cells decreased during aging [334]. These findings suggest that older patients with cancers such as lymphoma, glioblastoma and leukemia may be less responsive to immunotherapy.

Additionally, within the aged mouse model TME, CD8+ T cells displayed a tendency for exhaustion, and IFN-γ levels were significantly decreased; similarly, in aged patients with triple-negative breast cancer, IFN-γ gene expression levels were found to be decreased. Inflammation of the TME mediated by IFN-γ in aged tumor-bearing mice significantly increased the sensitivity of the mouse responses to ICIs [335]. Considerable high-quality evidence and substantial published datasets have confirmed that currently available ICIs show high efficacy in older adults [336–363] (Table 4); however, some of these findings are controversial and because of a lack of enrolled aged patients (maybe ≥ 65 years) in clinical trials, to determine whether the investigated treatments show clinical benefits or whether toxicity is increased in elderly patients, more study is needed [305, 364]. Patients older than 60 years are likely to respond more efficiently to anti-PD-1 immunotherapy, and the likelihood of eliciting a response to anti-PD-1 increases with age. These findings have been recapitulated in young and aged melanoma mouse models.

**Table 4:** Summary of key findings from main phase III randomized controlled trials administrating immunotherapy on the elderly

Trial name	Year	Intervention and control settings	Participants’ characteristics	Key results and findings
*Malignant melanoma*				
dacarbazine + ipilimumab versus dacarbazine + placebo (NCT00324155) [338]	2011	First line dacarbazine + ipilimumab versus dacarbazine	N = 502; median age = 57 yr; 65 years: 32%	OS ≥ 65 yr, HR: 0.99 (0.56–1.25); OS < 65 yr, HR: 0.67 (0.40–0.87)
KEYNOTE-006 (NCT01866319) [339]	2015	First line or pretreated pembrolizumab versus ipilimumab	N = 834; median age = 62 yr; 65 years: 43%	OS ≥ 65 yr, HR: 0.56 (0.36–0.87); OS < 65 yr, HR: 0.65 (0.44–0.95)
CheckMate 066 (NCT01721772) [340]	2014	First line nivolumab versus dacarbazine	N = 418; median age = 65 yr; 65 years: 52%	OS ≥ 75 yr, HR: 0.25 (0.10–0.61); OS 65–74 yr, HR: 0.44 (0.24–0.81); OS < 65 yr, HR: 0.52 (0.32–0.85)
CheckMate 067 (NCT01844505) [341]	2017	First line nivolumab + ipilimumab versus ipilimumab versus nivolumab	N = 945; median age = 60 yr; 65 years: 40%	OS ≥ 65 yr, HR: 0.69; OS < 65 yr, HR: 0.48 (n + i vs. i); OS ≥ 65 yr, HR: 0.96; OS < 65 yr, HR: 0.78 (n + i vs. n); OS ≥ 65 yr, HR: 0.71; OS < 65 yr, HR: 0.62 (n vs. i)
CheckMate 238 (NCT02388906) [342]	2017	Adjuvant nivolumab versus ipilimumab	N = 906; median age = 55 yr; 65 years: 26%	RFS ≥ 65 yr, HR: 0.66 (0.45–0.97); RFS < 65 yr, HR: 0.65 (0.51–0.84)
KEYNOTE-054 (NCT02362594) [343]	2018	Adjuvant pembrolizumab versus placebo	N = 1019; median age = 54 yr; 65 years: 25%	RFS ≥ 65 yr, HR: 0.55 (0.32–0.93); RFS < 65 yr, HR: 0.57 (0.41–0.80)
*Non-small cell lung cancer*				
KEYNOTE-024 (NCT02142738) [344]	2016	First line, TPS > 50%, pembrolizumab versus platinum-based chemotherapy	N = 305; median age = 65 yr; 65 years: 54%	OS ≥ 65 yr, HR: 0.64 (0.42–0.98); OS < 65 yr, HR: 0.60 (0.38–0.96)
KEYNOTE-042 (NCT02220894) [345]	2019	First line, TPS > 1%, pembrolizumab versus platinum-based chemotherapy	N = 1274; median age = 63 yr; 65 years: 45%	OS ≥ 65 yr, HR: 0.82 (0.66–1.01); OS < 65 yr, HR: 0.81 (0.67–0.98)
KEYNOTE-010 (NCT01905657) [346]	2016	Pretreated, pembrolizumab versus docetaxel	N = 1034; median age = 63 yr; 65 years: 41%	OS ≥ 65 yr, HR: 0.76 (0.57–1.02); OS < 65 yr, HR: 0.63 (0.50–0.79)
CheckMate 017 (NCT01642004) [347]	2015	Pretreated squamous, nivolumab versus docetaxel	N = 272; median age = 63 yr; 65 years: 44%	OS ≥ 75 yr, HR: 1.85 (0.76–4.51); OS 65–74 yr, HR: 0.56 (0.34–0.91); OS < 65 yr, HR: 0.52 (0.35–0.75)
CheckMate 057 (NCT01673867) [348]	2015	Pretreated non squamous, nivolumab versus docetaxel	N = 582; median age = 62 yr; 65 years: 42%	OS ≥ 75 yr, HR: 0.90 (0.43–0.87); OS 65–74 yr, HR: 0.63 (0.45–0.89); OS < 65 yr, HR: 0.81 (0.62–1.04)
OAK (NCT02008227) [349]	2017	Pretreated, atezolizumab versus docetaxel	N = 850; median age = 64 yr; 65 years: 47%	OS ≥ 65 yr, HR: 0.66 (0.52–0.83); OS < 65 yr, HR: 0.80 (0.64–1.00)
PACIFIC (NCT02125461) [350]	2017	Adjuvant concurrent chemoradiotherapy followed by durvalumab versus placebo	N = 709; median age = 64 yr; 65 years: 45%	OS ≥ 65 yr, HR: 0.76 (0.55–1.06); OS < 65 yr, HR: 0.62 (0.44–0.86)
KEYNOTE-189 (NCT02578680) [351]	2018	First line non squamous, cisplatin or carboplatin + pemetrexed + pembrolizumab versus cisplatin or carboplatin + pemetrexed	N = 616; median age = 64 yr; 65 years: 49%	OS ≥ 65 yr, HR: 0.64 (0.43–0.95); OS < 65 yr, HR: 0.43 (0.31–0.61)
KEYNOTE-407 (NCT02775435) [352]	2018	First line squamous, carboplatin + paclitaxel + pembrolizumab	N = 559; median age = 65 yr; 65 years: 55%	OS ≥ 65 yr, HR: 0.74 (0.51–1.07); OS < 65 yr, HR: 0.52 (0.34–0.80)
CheckMate 227 (NCT02477826) [353]	2019	First line, TPS > 1%, nivolumab + ipilimumab versus platinum-based chemotherapy	N = 1189; median age = 64 yr; 65 years: 49%	OS ≥ 75 yr, HR: 0.92 (0.57–1.48); OS 65–74 yr, HR: 0.91 (0.70–1.19); OS < 65 yr, HR: 0.70 (0.55–0.89)
CheckMate 9LA (NCT03215706) [354]	2021	First line nivolumab + ipilimumab + platinum-based chemotherapy versus platinum-based chemotherapy	N = 719; median age = 65 yr; 65 years: 51%	OS ≥ 75 yr, HR: 1.21; OS 65–74 yr, HR: 0.62; OS < 65 yr, HR: 0.61
IMpower150 (NCT02366143) [355]	2018	First line or post-TKI non squamous carboplatin + paclitaxel + bevacizumab + atezolizumab versus carboplatin + paclitaxel + bevacizumab	N = 800; median age = 63 yr; 65 years: 45%	PFS ≥ 75 yr, HR: 0.78; PFS 65–74 yr, HR: 0.52; PFS < 65 yr, HR: 0.65
IMpower010 (NCT02486718) [356]	2021	resected IB-IIIA with chemotherapy followed by atezolizumab versus BSC	N = 1005; median age = 62 yr; 65 years: 67%	DFS ≥ 65 yr, HR: 0.76 (0.54–1.05); DFS < 65 yr, HR: 0.79 (0.61–1.03)
*Renal cell carcinoma*				
CheckMate 025 (NCT01668784) [357]	2015	Pretreated nivolumab versus everolimus	N = 821; median age = 62 yr; 65 years: 39%	OS ≥ 75 yr, HR: 1.23 (0.66–2.31); OS 65–74 yr, HR: 0.64 (0.45–0.91); OS < 65 yr, HR: 0.78 (0.60–1.01)
CheckMate 214 (NCT02231749) [358]	2018	First line nivolumab + ipilimumab versus sunitinib	N = 1096; median age = 62 yr; 65 years: 38%	OS ≥ 75 yr, HR: 0.97 (0.48–1.95); OS 65–74 yr, HR: 0.86 (0.58–1.27); OS < 65 yr, HR: 0.53 (0.40–0.71)
KEYNOTE-426 (NCT02853331) [359]	2019	First line pembrolizumab + axitinib versus sunitinib	N = 861; median age = 62 yr; 65 years: 38%	OS ≥ 65 yr, HR: 0.59 (0.36–0.97); OS < 65 yr, HR: 0.47 (0.30–0.73)
JAVELIN 101 (NCT02684006) [360]	2019	First line avelumab + axitinib versus sunitinib	N = 886; median age = 61 yr; 65 years: 38%	PFS ≥ 65 yr, HR: 0.70 (0.49–0.99); PFS < 65 yr, HR: 0.68 (0.54–0.87)
*Urothelial carcinoma*				
KEYNOTE-045 (NCT02256436) [361]	2017	Pretreated pembrolizumab versus investigator choice chemotherapy	N = 542; median age = 66 yr; 65 years: 58%	OS ≥ 65 yr, HR: 0.76 (0.56–1.02); OS < 65 yr, HR: 0.75 (0.53–1.05)
IMvigor-130 (NCT02807636) [362]	2020	First line platinum-based chemotherapy + atezolizumab versus platinum-based chemotherapy	N = 1231; median age = 68 yr; 65 years: 63%	PFS ≥ 65 yr, HR: 0.80 (0.66–0.97); PFS < 65 yr, HR: 0.82 (0.63–1.06)
JAVELIN 100 (NCT02603432) [363]	2020	first line maintenance avelumab + BSC versus BSC	N = 700; median age = 69 yr; 65 years: 66%	OS ≥ 65 yr, HR: 0.63 (0.47–0.83); OS < 65 yr, HR: 0.79 (0.55–1.15)

*OS* overall survival, *HR* hazard ratio, *RFS* recurrence-free survival, *PFS* progression-free survival, *DFS* disease-free survival, *TPS* tumor proportion score, *TKI* tyrosine kinase inhibitor, *BSC* best supportive care, *yr* year (Mainly from 10.1200/JCO.21.00138)

Moreover, research on TME immune cell subtypes revealed that aged mice showed significantly increased CD8+ T-cell-to-Treg cell ratios, indicating that they carried more immunogenic tumors [305]. The depletion of Treg cells via anti-CD25 therapy significantly increased the anti-PD-1 response in young mice [305], and Treg cell depletion in aged melanoma mouse models was ineffective in inducing antitumor immunity but completely decreased tumor growth in young mice [308]. However, other studies have shown contradictory findings. For example, in one study, anti-PD-L1 treatment of B16 melanomas exhibited substantial efficacy only in young mice, but combination therapy with anti-PD-L1 and anti-CTLA4 antibodies showed partial efficacy in aged mice [365]. Some retrospective studies on melanoma [364] and non-small cell lung cancer (NSCLC) [366] in which either anti-PD-1 or anti-PD-L1 was administered showed little difference in overall survival (OS), progression-free survival (PFS) or toxicity between age groups. Because the diversity in immune cell profiles, infiltration status, and activity across various tumor models as well as in the TME at different tissue sites is large, the immunotherapeutic response likely differs marked in young versus aged patients. Genetic and environmental diversity in different populations also limits collective analyses across various racial and ethnic backgrounds. Given the discrepancy found when targeting one immune checkpoint compared with another in aged models, tailoring specific immunotherapeutic treatments to aged patients may be warranted. Importantly, some groups are focusing on seeking strategies for recruiting older patients with cancer for clinical trials [367]. These strategies, along with a focus on furthering the understanding of age-related changes at the molecular level in TMEs and premetastatic niches, may be critical in efficiently predicting patient responses across a wide range of cancers. Considering these possibilities, we may also identify other avenues for effective cotargeting of tumor-promoting immune cell subpopulations.

## Conclusions and further perspectives

Recent studies have suggested that the aging TME may exert dramatic effects on tumor progression. Normal age-related changes in stromal and immune populations may function together to drive the progression of tumor cells from an initial or slow-growing state to a highly aggressive and metastatic disease state. The outcomes of these changes involve variations in secreted factors, changes in the biophysical architecture of the TME, and even changes on a macroscopic level, such as the breakdown in vasculature integrity [368, 369]. Several challenges remain that need to be resolved in the future. (1) There is a lack of drugs that are highly effective in inducing senescence in a high proportion of cancer cells. In addition, drugs need to show a preferential affinity for cancer cells over normal cells, as inducing senescence in normal tissues can cause detrimental side effects [88]. (2) We need to establish more gold standard signatures and biomarkers for identifying the senescent state. No obvious biomarker can be measured to unambiguously discriminate between senescence and other growth-arresting states [86]. There have been several multi-gene signatures to identify senescence in primary cells. A panel of cancer cells have been selected to identify the SENCAN classifier for cancer senescence [86]. In vivo, investigators should use galacto-conjugated fluorescent nanoparticles to detect senescent cells. This method has been tested in models of chemotherapy-induced senescence [370, 371]. The noninvasive imaging system could be ideal for measuring the efficacy of senescence induction in cancer cells. Radioactive β-gal positron emission computed tomography (PET) tracer seems to be feasible [372]; however, β-gal-based screening strategy might be unpowered to detect all senescent cells accurately. On the one hand, cells from some tissue types do not induce SA-β-gal activity when they turn to senescent [8], on the other hand, macrophages may also be able to exhibit increased SA-β-gal activity [373, 374], consequently, false positives may result from macrophages inside inflamed tumor tissues. Other noninvasive methods, for example, oxylipin biosynthesis may also help to detect clearance of senescent cells [375]. Furthermore, other biomarkers that can be potentially used in noninvasive approaches to detect senescent cancer cells should be similarly established [376, 377]. Researchers need to explore whether these markers can be used to detect senescent cancer cells in different contexts, such as cells with different genetic backgrounds, derived from different tissue types or with senescence induced by different agents [378]. (3) The next challenge is that there is still no unique senescence-based therapy due to tumor heterogeneity. Intratumoral heterogeneity leads to varying drug responses that may limit the effectiveness of senescence induction within tumors. Senescent cells can spread the senescent phenotype through the SASP to the surrounding non-senescent cells within tumors [101], which will sensitize non-senescent cancer cells to senolytic treatments. Additionally, such bystander effects could be fortified by the local TME shaped by SASP of the senescent cells. Further efforts to understand senescence-based therapy outcomes may overcome tumor heterogeneity and guide the timing of personalized treatments [101, 379]. Most senolytic drugs were developed with the aim to reverse the effects of aging and were consequently tested mostly on primary cells. In a study, the commonly used senolytic (navitoclax) on a panel of senescent cancer cells showed that it has widely variable activity as a senolytic [86]. Beyond identifying absolute biomarkers for the senescent state, the field is also in need of a druggable and broadly present vulnerability of senescent cancer cells. Novel CRISPR-Cas9-based genetic screening platform allows for the performance of drop-out screens to identify new senolytic targets on the genome scale. If universal vulnerabilities of senescent cancer cells exist, unbiased genetic screens should allow the identification. (**4**) There is still no abundant knowledge on how the SASP acquired by senescent cancer cells impacts the interaction between senescent cancer cells and immune system cells. Several early studies have pinpointed a synergy between senescence-promoting therapy and checkpoint immunotherapy [36, 37]. It is possible that not all pro-senescence therapies and not all cancer types will benefit from combination checkpoint immunotherapy. This possibility is supported by the substantial heterogeneity in SASP factors produced by different types of cells undergoing senescence [86]. It is important to ascertain when a SASP provokes an immune response that can be enhanced by checkpoint immunotherapy and when it does not. It is possible to use the so-called senomorphic drugs as the NF-κB-inhibiting drugs apigenin and kaempferol or the mTOR inhibitor rapamycin [380, 381], which can modify the SASP of senescent cells to become more responsive to checkpoint therapy clearance [382]. (5) We need to be significantly cautious in ablating senescent normal cells via anti-senescence therapies in aged individuals. In elderly individuals, senescent cells can constitute a high percentage of the net number of cells in some tissues and this may jeopardize tissue structural integrity or affect vascular endothelial cells, leading to blood–tissue barrier disorder that potentially leads to liver and perivascular tissue fibrosis and health collapse [383, 384]. This issue highlights the need for the development of cancer-selective senolytics.

In this article, we discuss the impact of aging on the TME from multiple perspectives and review treatments as well as recent clinical trials with data on elderly individuals. The effects of aging on tumors are two-sided. In the early stage of tumor formation, aging is often associated with tumor suppression. However, once a tumor progresses past a certain threshold, the tumor-suppressive mechanisms are exploited by tumors, which increases their malignancy. To some extent, these mechanisms exhibit a screening function for tumors. In addition, in special elderly groups, in addition to age-associated alterations at the local cellular and molecular levels, changes in organs may play an important role in the tumor microenvironment. Therefore, for tumor prevention and treatment in elderly people, in addition to focusing on existing treatment methods, the influence of various organs and biological systems needs to be comprehensively considered.

## Data Availability

The datasets used and/or analyzed during the current study are available from the corresponding author upon reasonable request.

## Abbreviations

ACD: Allergic contact dermatitis
ALT: Alternative lengthening of telomeres
CDK: Cyclin-dependent kinase
cGAS: Cyclic GMP-AMP synthase
CRP: C-reactive protein
CSF1R: CSF1 receptor
CTC: Circulating tumor cells
CTLA-4: Cytotoxic T lymphocyte-associated antigen 4
C/EBPα: CCAAT/enhancer-binding protein α
DDR: DNA damage response
DNFB: Dinitrobenzene
miRNAs: MicroRNAs
ECM: Extracellular matrix
EMT: Epithelial–mesenchymal transition
EPC: Endothelial progenitor cells
GEMMs: Genetically engineered mouse models
GM-CSF: Granulocyte–macrophage colony-stimulating factor
HDAC: Histone deacetylase
ICIs: Immune checkpoint inhibitors
IDO1: Indoleamine 2,3-dioxygenase 1
IGF1: Insulin-like growth factor 1
LAG-3: Lymphocyte activation gene 3
LINE-1: Long-interspersed element-1
MDSCs: Myeloid-derived suppressor cells
MSCs: Mesenchymal stem cells
Mo-MDSCs: Monocytic-MDSCs
NK cells: Natural killer cells
OIS: Oncogene-induced senescence
OS: Overall survival
PD-1: Programmed cell death 1
PD-L1: Programmed cell death ligand 1
PET: Positron emission computed tomography
PFS: Progression-free survival
PICS: PTEN-loss-induced cellular senescence
PML: Promyelocytic leukemia
QoL: Quality of life
ROS: Reactive oxygen species
SAE: Senescence activation enhancer
SASP: Senescence-associated secretory phenotype
SCC: Squamous cell carcinoma
STING: Stimulator of interferon genes
TAMs: Tumor-associated macrophages
TANs: Tumor-associated neutrophils
TERT: Telomerase reverse transcriptase
TIME: Tumor-immune microenvironment
TIS: Therapy-induced senescence
TLR8: Toll-like receptor 8
TME: Tumor microenvironment
TMMs: Telomere maintenance mechanisms
Treg: T-regulatory cells
T-SCE: Telomere sister chromatid exchange
UPR: Unfolded protein response
VEGF: Endothelial growth factor
VM: Vasculogenic mimicry
